# Breaking Barriers: A Future Perspective on Glioblastoma Therapy with mRNA-Based Immunotherapies and Oncolytic Viruses

**DOI:** 10.3390/vaccines12010061

**Published:** 2024-01-08

**Authors:** Alexandro Guterres, Paulo Niemeyer Soares Filho, Vivaldo Moura-Neto

**Affiliations:** 1 Laboratório de Hantaviroses e Rickettsioses, Instituto Oswaldo Cruz, Fundação Oswaldo Cruz (FIOCRUZ), Rio de Janeiro 21040-360, RJ, Brazil; 2 Laboratório de Tecnologia Imunológica, Instituto de Tecnologia em Imunobiológicos, Vice-Diretoria de Desenvolvimento Tecnológico, Bio-Manguinhos, Fundação Oswaldo Cruz (FIOCRUZ), Rio de Janeiro 21040-360, RJ, Brazil; 3 Instituto Estadual do Cérebro Paulo Niemeyer, Rio de Janeiro 20231-092, RJ, Brazil; niemeyer@globo.com (P.N.S.F.);; 4 Instituto de Ciências Biomédicas, Universidade Federal do Rio de Janeiro, Rio de Janeiro 21941-590, RJ, Brazil

**Keywords:** molecular therapy, mRNA-based immunotherapy, oncolytic virus, glioblastoma, cancer treatment, Zika virus, glioblastoma stem cells, oncolytic virotherapy

## Abstract

The use of mRNA-based immunotherapies that leverage the genomes of oncolytic viruses holds significant promise in addressing glioblastoma (GBM), an exceptionally aggressive neurological tumor. We explore the significance of mRNA-based platforms in the area of immunotherapy, introducing an innovative approach to mitigate the risks associated with the use of live viruses in cancer treatment. The ability to customize oncolytic virus genome sequences enables researchers to precisely target specific cancer cells, either through viral genome segments containing structural proteins or through a combination of regions with oncolytic potential. This strategy may enhance treatment effectiveness while minimizing unintended impacts on non-cancerous cells. A notable case highlighted here pertains to advanced findings regarding the application of the Zika virus (ZIKV) in GBM treatment. ZIKV, a member of the family Flaviviridae, shows oncolytic properties against GBM, opening novel therapeutic avenues. We explore intensive investigations of glioblastoma stem cells, recognized as key drivers in GBM initiation, progression, and resistance to therapy. However, a comprehensive elucidation of ZIKV’s underlying mechanisms is imperative to pave the way for ZIKV-based clinical trials targeting GBM patients. This investigation into harnessing the potential of oncolytic-virus genomes for mRNA-based immunotherapies underscores its noteworthy implications, potentially paving the way for a paradigm shift in cancer treatment strategies.

## 1. Introduction

Brain tumors are a critical area of study within the realm of medical research and neuro-oncology. These neoplastic growths, originating from abnormal cell proliferation in the brain or its adjacent tissues, encompass a wide spectrum of biological behaviors and clinical manifestations [1]. These tumors can be broadly classified into two categories: primary brain tumors, which develop directly from brain cells, and secondary brain tumors, also known as metastatic tumors, which originate from cancer cells that have spread to the brain from other parts of the body [2]. Brain tumors pose complex medical challenges because of their intricate location in the central nervous system and their potential to affect various cognitive, motor, and sensory functions [3,4].

Glioblastoma multiforme (here termed GBM), one of the most aggressive and lethal primary brain tumors, poses a significant challenge in the field of neuro-oncology [5,6]. Its intricate biological characteristics, including rapid growth, high invasiveness, and resistance to conventional therapies contribute to its notorious reputation [7]. The presence of a family of proteins that are resistant and extrude drugs (multidrug resistance MDR) such as temozolomide contributes crucially to tumor survival. The genome landscape and heterogeneity of GBM further complicate treatment, necessitating an in-depth exploration of its molecular underpinnings [8,9]. With advances in genome profiling and molecular analysis, researchers are uncovering key genetic alterations and signaling pathways that drive GBM progression [8]. In light of the complex challenges posed by glioblastoma, effective treatment strategies remain elusive. GB, characterized by its aggressive and infiltrative nature, presents a formidable obstacle to conventional therapeutic approaches. The tumor’s location in the intricate neural network of the central nervous system further complicates interventions, limiting the feasibility of complete surgical resection and fostering resistance to standard treatments [10,11].

**Figure 1:**
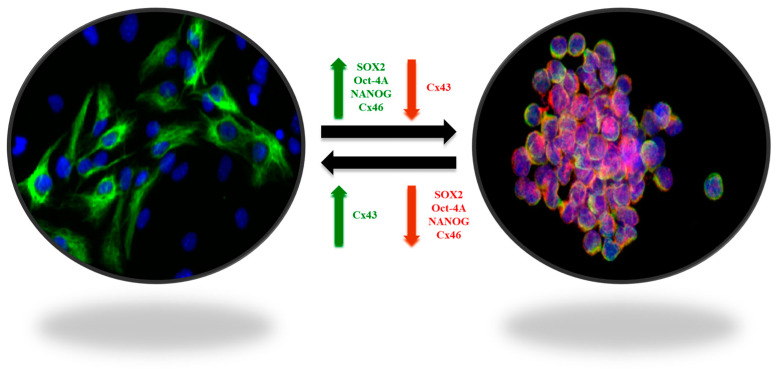
Plasticity of GBM stem-like cells. The balance between self-renewal and differentiation properties is dynamic and controlled by interactions of tumor cells with their microenvironment. The stem-like cell markers SOX2, OCT-4A, Nanog, and connexin 46 (Cx46), involved in cell-to-cell communication processes, are overexpressed in GSCs (green arrow), whereas connexin 43 (Cx43) is downregulated (red arrow). In contrast, in non-GSCs, SOX2, OCT-4A, Nanog, and Cx46 are downregulated (red arrow), whereas Cx43 in overexpressed (green arrow) [15].

Despite advances in radiation therapy, chemotherapy and targeted agents, inherent biological heterogeneity, intricate cellular interactions, and the presence of a protective blood–brain barrier collectively thwart successful treatment outcomes [12,13,14]. The relentless recurrence of glioblastoma and the limited success in significantly extending patient survival underscore the urgent need for innovative and multidimensional therapeutic paradigms to combat this formidable malignancy. The difficulty of treating GBM lies in its cellular heterogeneity, which together with the surrounding microenvironment contributes to the tumor progression and “protection”. With respect to GBM, the singular contribution of their stem cells allows growth, diffusion, and establishment of the tumor in a second or third region of the brain, leading to recurrence. The central point of attack for this tumor could be these tumor stem cells [15]. These stem cells diffuse within the brain, generating new foci of tumorigenesis in different regions. Tumors are currently understood to derive originally from the monoclonal expansion of stem cells, clonal selection due to mutations, or chromosome instability [16]. Furthermore, expanding stem cells can differentiate into tumor cells or from these become stem cells, a transition followed by expression of markers such as indicated below in both phases of these changes [15] (Figure 1). Additionally, studies aimed at identifying potential antigens in GBM for the development of advanced RNA-based therapies have identified numerous distinct antigen sets, thereby augmenting the challenge of comprehensive treatment [17].

## 2. Oncolytic Virotherapy

Oncolytic virotherapy, an expanding frontier in cancer treatment, revolves around the strategic use of viruses as therapeutic agents to target and eliminate malignant cells [18]. This innovative approach capitalizes on the inherent ability of certain viruses to selectively infect and replicate in cancer cells, leading to their destruction while sparing healthy tissues. Engineered virotherapies leverage modifications that enhance tumor selectivity, replication efficiency, and immune activation, fostering a multifaceted assault on the tumor microenvironment [19,20]. The viral oncolysis, coupled with the potential for tumor-specific antigen release, fuels antitumor immune responses, thereby amplifying the therapeutic impact [21]. A spectrum of viruses, including adenoviruses, herpesviruses, and measles viruses, among others, have yielded promising outcomes in preclinical and clinical settings. Oncolytic virotherapy, bridging virology and oncology, harnesses viruses as powerful allies in the ongoing quest to develop more effective, targeted, and personalized strategies to combat cancer [19].

A comprehensive review of clinical trials reveals a total of 196 registered studies (https://clinicaltrials.gov/, accessed on 15 November 2023) employing approaches based on oncolytic viruses (Supplementary Table S1). Twenty-eight of them focus specifically on the use of oncolytic viruses for treatment of tumors of the central nervous system, especially glioblastomas. Most of these studies are in stage 1 or 2, indicating the active exploration and early assessment of these novel strategies, using a variety of methods. It should be noted that 12 of the 28 studies are conducted utilizing Adenovirus. Some employ a single oncolytic virus as the therapeutic intervention, while others adopt a more complex approach. This involves combining the oncolytic virus with established pharmaceutical agents, such as temozolomide, which is the current gold standard in clinical practice. The combination of these innovative viral therapies with existing medical approaches reflects the multifaceted nature of ongoing efforts in advancing the field of oncolytic virus-based treatments for central nervous system tumors (Table 1).

The field of neuro-oncology research has seen promising growth with the emergence of clinical trials investigating adenovirus-based therapies for brain tumors [22]. Adenoviruses, renowned for their broad cell infectivity, have been strategically repurposed to capitalize on their oncolytic potential against brain malignancies. Engineered through genetic modifications, adenoviruses, exemplifying their prowess, selectively replicate in tumor cells by capitalizing on their distinct signaling pathways and inducing targeted cell death [23,24]. Furthermore, adenoviruses can be equipped with therapeutic transgenes to increase their antitumor properties. Many clinical trials, meticulously documented on platforms such as ClinicalTrials.gov, have examined the safety and therapeutic efficacy of adenovirus-based interventions in patients with brain tumors, including glioblastoma and various intracranial malignancies (Table 1). These trials have used an array of treatment strategies, ranging from localized intratumor administration of replication-competent adenoviruses to the delivery of engineered adenoviral vectors encoding tumor-suppressive genes or immune-boosting agents [24,25]. Preliminary results have indicated tumor regression, extended survival rates, and potential synergistic interactions with complementary therapeutic modalities. The multifaceted approach of adenovirus-based therapies, combining oncolytic and immunomodulatory mechanisms, offers a promising avenue to surmount the complex hurdles posed by brain tumors and transcend the confines of traditional treatments [26,27]. As research in this area evolves, clinical trials using adenovirus-based strategies may result in innovative therapeutic initiatives for brain tumors [28,29]. These trials, in addition to underscoring the adaptability of adenoviruses as versatile tools in the realm of neuro-oncology, spark a renewed sense of optimism for the development of more efficacious and individualized treatments, rekindling hope for patients contending with these formidable malignancies [26,30,31].

The selection of specific virus entities for oncolytic virotherapy involves a careful consideration of the potential advantages and disadvantages inherent to each candidate. Adenoviruses, for instance, are renowned for their efficient gene delivery capabilities and well-established safety profiles, making them attractive candidates for oncolytic applications. However, concerns about preexisting immunity and limited capacity for sustained replication may impact their therapeutic efficacy. Herpes simplex viruses (HSV), including those engineered such as G47, exhibit strong lytic activity against tumor cells and have the advantage of prolonged replication within the neoplastic environment. Nevertheless, the potential for neurotoxicity and the development of antiviral resistance are noteworthy considerations [32]. RNA viruses, such as measles viruses, are characterized by robust oncolytic potential and the ability to trigger potent antitumor immune responses. However, their rapid clearance by the immune system and the risk of toxicity poses challenges [33]. A careful evaluation of these advantages and disadvantages is essential for informed decision making in the development of oncolytic virotherapies, with the aim of optimizing their safety and efficacy profiles for cancer treatment [34]. Ensuring the stability and activity of oncolytic viruses is paramount for their successful clinical utilization in cancer therapy. The imperative for long-term storage stability in clinical settings underscores the importance of formulation design in preserving the efficacy of oncolytic viruses. Some authors address critical degradation factors and their mechanisms, including pH variations, thermal stress, freeze–thaw damage, surface adsorption, and oxidation, which oncolytic viruses encounter during storage [35].

While the majority of clinical studies are currently in phase II [36], a notable exception is observed in Japan where the Ministry of Health, Labour and Welfare (MHLW) granted conditional approval to Daiichi Sankyo’s oncolytic virotherapy, Delytact (G47∆; teserpaturev), for the treatment of malignant glioma in 2021. This groundbreaking decision not only marks the first approval of Delytact, but also signifies the inaugural approval of an oncolytic virus treatment for brain cancer. Delytact, a collaborative development of Daiichi Sankyo and the University of Tokyo’s Institute of Medical Science, is a triple-mutated, replication-conditional herpes simplex virus type 1 (HSV-1) engineered to selectively replicate within cancer cells. Referred to as oncolytic immunotherapy, these engineered viruses exhibit selective replication in tumor cells until cellular lysis occurs, followed by the release of additional viruses targeting successive tumor cells. The approval, valid for seven years, is based on data from a single-arm, investigator-initiated Phase II study in Japan in patients with residual or recurrent glioblastoma, where Delytact met the primary endpoint of a one-year survival rate [37,38].

### Zika Virus and Glioblastoma

Exploration of the oncolytic potential of the Zika virus (ZIKV) against GBM is a novel and promising frontier in the field of cancer research. Originally associated with neurological complications, the ZIKV has recently attracted attention for its unique ability to selectively target and infect neural progenitor cells, leading to their destruction [39]. In the context of GBM, which are characterized by their aggressive and infiltrative nature, the ZIKV shows an intriguing potential to preferentially infect and eliminate glioblastoma stem cells (GSCs), a subset of cells implicated in tumor recurrence and resistance to therapies [40,41]. Preliminary studies have indicated that the Zika virus’s oncolytic effect could be harnessed to selectively target glioblastoma cells, offering a highly targeted therapeutic approach that holds promise for circumventing the challenges associated with conventional treatments [42].

Researchers have explored the application of oncolytic virus therapy in combatting glioblastoma, focusing on Zika virus (ZIKV), a flavivirus acknowledged for its propensity to elicit cell death and neural precursor cell differentiation during fetal development [43]. A study revealed the distinctive predilection for infecting and eradicating GSCs, a preference that remained conspicuously absent when confronted with differentiated tumor progeny or normal neuron cells [44]. Importantly, this GSC-targeted impact was not a universal trait among neurotropic flaviviruses, as the West Nile virus showed indiscriminate cytotoxicity to both malignant and normal neural cells. In particular, ZIKV caused a potent and specific GSC depletion in cultures and organoid models derived from patient samples. The translational potential shown by these findings was supported through in vivo experiments, where mice afflicted with glioblastoma showed significantly prolonged survival rates and enhanced overall survival upon intracranial inoculation with a mouse-adapted ZIKV strain [44]. These outcomes collectively suggest that ZIKV is an oncolytic virus, uniquely equipped to selectively target GSCs.

One study established that ZIKV selectively targets GBM stem cells, consequently reducing the mortality associated with gliomas in mice [45]. The researchers further embarked on a comprehensive assessment of the underlying immunological mechanisms governing the protective effects induced by ZIKV against GBM. The introduction of ZIKV into the cerebral tumor microenvironment engendered a marked increase in the infiltration of CD8+ T cells and myeloid cells. The indispensability of CD8+ T cells in ZIKV-mediated tumor eradication was substantiated by the attenuation of survival benefits after depletion of these cells. Intriguingly, the juxtaposition of ZIKV with anti-PD-1 antibody monotherapy produced a synergistic enhancement of tumor survival rates, surpassing the incremental effect of monotherapy alone. ZIKV-induced tumor clearance exhibited persistent protection against syngeneic tumor rechallenge, a response that was reliant on the presence of CD8+ T cells. In addressing safety concerns, the researchers successfully engineered an immune-sensitized strain of ZIKV, which showed efficacy either as a monotherapy or in tandem with immunotherapeutic interventions. Therefore, the therapeutic potential of oncolytic ZIKV treatment can be harnessed synergistically with immunotherapies, indicating the prospect of developing combination treatment regimens tailored to the specific requirements of adult patients afflicted with GBM [45]. This study improved our understanding of the intricate interplay between viral oncolysis and immune response, paving the way for novel therapeutic strategies in the realm of GBM treatment.

In one study, the functional implications of several non-structural proteins were examined in the context of tumor suppression. Specifically, the roles of NS1, NS3, NS4B, and NS5 were investigated in the human glioma cell line U87 [46]. Notably, the inhibitory effect on proliferation, migration, and invasion of U87 cells was significant with NS5. In vivo experiments showed that the expression of NS5 effectively suppressed the tumorigenic potential of mouse GL261 glioma cells. These findings collectively contributed pivotal insights into the potential of leveraging the oncolytic properties of the Zika virus, particularly through NS5, as a promising avenue for the treatment of glioma [46].

In a recent study, investigators presented an interesting case involving a patient with glioblastoma who underwent the conventional standard-of-care therapy, including surgical resection, radiotherapy, and temozolomide administration [47]. Interestingly, at about the same time as the tumor-mass resection, the patient was simultaneously afflicted with a clinical diagnosis reminiscent of an arbovirus-like infection, which transpired during a Zika virus outbreak in Brazil. After the successful resolution of the infection, a profound regression of the glioblastoma occurred, accompanied by the absence of any recurrence. This remarkable clinical response was consistently sustained, enduring for a remarkable span of 6 years (7 years now) following the initial diagnosis of glioblastoma [47]. ZIKV has shown oncolytic capacity, able to infect and trigger cell death mainly in the glioblastoma stem-like cell populations (Sox2+Ki67+) in vitro and in vivo, in animal models [48,49,50]. In conclusion, the oncolytic ability of flaviviruses could be explored as a novel brain-cancer therapy to reduce the glioblastoma stem-like cells and therefore prolong the patient’s lifespan.

The utilization of live oncolytic viruses for cancer treatment presents both a promising therapeutic potential and notable safety considerations. While live oncolytic viruses have the capacity to specifically target and destroy cancer cells, their inherent ability to replicate and spread within the body raises concerns about potential unintended consequences [34]. It is worth noting, however, that for the majority of adult patients, Zika virus infection has been demonstrated to pose minimal danger [51,52]. The very attribute that underpins their efficacy—the ability to infiltrate, replicate, and propagate within the host organism—conjures up a complex interplay of safety concerns. The possibility that rampant viral replication may transcend its intended battlefield—the tumor microenvironment—and encroach upon healthy tissues, causing inadvertent destruction and ensuing systemic effects, illustrates the gravity of this concern. The risk of uncontrolled viral replication, leading to tissue damage and systemic effects, requires a meticulous evaluation of the balance between therapeutic benefit and potential harm [34,53]. Furthermore, the immunogenicity of live viruses can trigger immune responses that may compromise the virus’s therapeutic effectiveness or result in adverse reactions [54]. Amid these considerations surrounding live oncolytic virus therapy, there remains the potential to harness the therapeutic potential of viruses while circumventing the challenges of uncontrolled replication and off-target effects. Self-amplifying RNA technology offers an innovative solution that capitalizes on the versatility of genetic manipulation to orchestrate a finely tuned immune response, a compelling alternative to live-virus therapies.

## 3. Oncolytic Virotherapy and Self-Amplifying RNA Technology

Groundbreaking advances in cancer therapeutics include the fusion of oncolytic virotherapy and self-amplifying RNA technology. These cutting-edge methods not only harness the potential of mRNA-based platforms within immunotherapies but also may alleviate the risks inherent in employing live viruses in the treatment of cancer [55,56]. In recent years, mRNA, the intermediary between DNA and protein synthesis, has been ingeniously repurposed to elicit robust immune responses against a range of diseases. This innovation capitalizes on the inherent ability of mRNA to encode antigenic information, thereby enabling the precise design and production of immunogenic proteins within host cells [57]. This revolutionary approach has opened avenues to address the complex challenges posed by various diseases, prominently including cancer. In the context of cancer, mRNA technology offers unprecedented opportunities for personalized and targeted therapeutic strategies [58]. By leveraging the customizable nature of mRNA sequences, researchers can precisely tailor therapeutic agents to trigger immune responses against cancer-specific antigens. This has inaugurated a new era in cancer immunotherapy, where mRNA-based vaccines and therapeutics have immense promise in harnessing the immune system to selectively recognize and eliminate malignant cells while minimizing damage to healthy tissues [59,60,61].

The use of self-amplifying RNA (saRNA) technology represents a cutting-edge approach in the field of nucleic acid-based therapeutics. This innovative technology involves the design and engineering of RNA molecules capable of not only encoding therapeutic proteins but also facilitating their own replication within host cells [62,63]. The constructs of saRNA are relatively large, with reports indicating sizes of up to 15,000 nucleotides [63,64]. Unlike conventional mRNA, saRNA possesses elements derived from positive-sense RNA viruses, such as alphaviruses. SaRNA vaccines employ RNA-dependent RNA polymerases (RDRP) derived primarily from RNA viruses, predominantly alphaviruses, to facilitate the amplification of the delivered RNA, thus augmenting the production of antigen proteins. In addition to the typical mRNA components, saRNA encompasses substantial open reading frames (ORF) encoding the elements necessary for RDRP, comprising nonstructural proteins 1-4 (nsP1-4), and the gene of interest, all under the control of a subgenomic promoter. The nsP1, 2, 3, and 4 sequences govern the synthesis of proteins responsible for mRNA capping, NTPase/Helicase/protease, macrodomain, and RDRP, respectively [65,66]. Upon cellular uptake, the saRNA enters the host cell’s cytoplasm, where it utilizes the cellular machinery to both translate the therapeutic protein of interest and replicate its own RNA. This inherent self-amplification feature results in a robust and prolonged protein expression, enhancing the efficiency of the therapeutic intervention [63,67]. Alphaviruses, for instance, can accumulate an estimated 10^6^ RNA copies per cell [68]. The expression in saRNA technology is considerably more enduring compared to the conventional mRNA platform, with a persistence typically extending over several days. This prolonged duration is attributed to the inherent characteristics of saRNA, making it particularly suitable for applications in vaccine development and cancer therapy [66]. Additionally, the multifunctional composition permits the simultaneous encoding of multiple antigens or immunomodulatory elements within a single construct, thus engendering a more nuanced and versatile immune response. The inherent capability of saRNA to accommodate multifunctional compositions is a pivotal point in the advancement of genetic therapeutics [62,63,69]. This attribute confers on saRNA a unique versatility, enabling the concurrent encoding of several antigens or immunomodulatory elements within a single construct. By orchestrating such complex genetic architectures, saRNA can trigger a dynamic and multifaceted immune response, heightening the efficacy of disease intervention [62,70,71,72]. This nuanced approach has the potential to stimulate the activation of various components of the immune system, including cellular and humoral responses, while simultaneously tailoring the immune environment to target specific pathogenic challenges. In addition, the multifunctional capacity of saRNA not only amplifies the immunity stimulating potential but also streamlines the administration process, as multiple therapeutic components can be delivered simultaneously [64,73,74].

**Figure 2:**
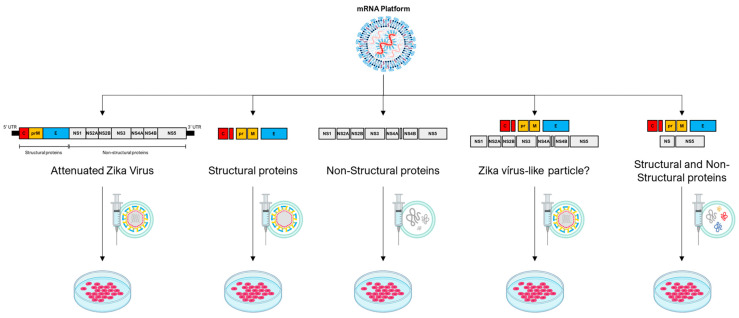
The mRNA platform, primarily self-amplifying RNA technology, demonstrates a broad range of possibilities for potential therapeutic constructs employing the genome of an oncolytic virus, represented here by the Zika virus. In the first construct, the entire genome of the Zika virus is used with modifications that make it attenuated. In the second construct, structural proteins of the Zika virus are employed, resulting in the formation of a non-infectious empty particle. In the third construct, non-structural proteins are used to create a cluster of proteins with potential oncolytic properties. In the fourth construct, a combination of two Zika virus polyproteins is proposed, one containing structural and the other non-structural elements, potentially forming an infectious viral particle. In the fifth construct, it is possible to combine some structural and non-structural proteins to form a cluster of oncolytic proteins or even a non-infectious particle containing both structural and select non-structural proteins.

Strategic integration of the saRNA multifunctional composition for use in oncolytic virotherapy is a remarkable advancement, with transformative implications for antitumor interventions. By capitalizing on the unique capability to accommodate the simultaneous encoding of various antigens or immunomodulatory elements within a unified construct [62,63,70], the potential of saRNA-driven oncolytic virotherapy is poised for a paradigm shift. Through this approach, saRNA can be tailored to elicit an intricately orchestrated and adaptable immune response against malignancies [64,74]. For instance, this strategy could encompass the delivery of a composition of structural proteins that assemble into nonreplicating virus-like particles, potentially mimicking viral presentation while mitigating replication risk. Alternatively, a hybrid composition could be devised, blending structural and non-structural proteins with intrinsic oncolytic capabilities (Figure 2). This multifaceted approach enables the therapeutic construct to exert concerted antitumor effects, utilizing multiple modalities of immune engagement while targeting distinct aspects of tumor biology. Consequently, this innovative integration embodies a convergence of precise genetic manipulation and oncolytic viral vectors, culminating in a finely tuned arsenal capable of triggering an adaptable, versatile, and potentially curative immune response against cancer.

Both DNA and RNA viruses induce a metabolic shift and exploit cellular mechanisms, including the cell cycle and signaling pathways, within host cells to optimize viral production [75]. The genomic structure of viruses encompasses two transcriptional units responsible for encoding structural and non-structural proteins. Structural proteins, integral to viral particles, perform vital functions such as cellular recognition, fusion, entry, or replication [76,77]. On the other hand, non-structural proteins (NS) are implicated in diverse cellular hijacking processes, such as the formation of inclusion bodies, interaction with the cytoskeleton, induction of apoptosis, and modulation of autophagy [78,79]. Although the functional roles of all NS proteins remain incompletely understood, some actively participate in the virus’s replication and latency cycle. Varied viral proteins follow distinct yet converging pathways to selectively modulate cellular events like the cell cycle and apoptosis in human cancer cells, leveraging existing aberrations for targeted effects [80]. Leveraging saRNA technology makes it possible to seamlessly integrate structural proteins, which exhibit high affinity for tumor cells (in terms of cell recognition, fusion, and entry), with non-structural proteins known for their robust oncolytic capabilities (Table 2). This strategic fusion enables a nuanced and targeted approach, capitalizing on the distinct strengths of each protein category to amplify the efficacy of the oncolytic virus against cancer cells. This genetic manipulation opens avenues to leverage the best attributes of diverse oncolytic viruses, regardless of their genomic types.

The utilization of various viral genomes within the saRNA platform requires a careful consideration of distinct mechanisms associated with different viral types. The selection of viral genomes plays a pivotal role in defining the characteristics and potential applications of saRNA. For dsDNA viruses, such as adenovirus, vaccinia virus, and herpesvirus, saRNA sequences must encompass the essential elements needed for these processes. The design should consider encoding components like need to include promoters that facilitate the transcription of viral genes and regulatory elements to control the timing and efficiency of gene expression [96,97,98]. This involves designing saRNA sequences that mimic the necessary viral components responsible for initiating these processes, ensuring compatibility with the host cell machinery.

On the other hand, for ssRNA viruses, whether positive-sense (e.g., coxsackievirus, Seneca Valley virus, poliovirus) or negative-sense (e.g., measles virus, Newcastle Disease virus, vesicular stomatitis virus), the saRNA platform must be tailored to replicate the specific genomic features of these viruses. In positive-sense ssRNA viruses, saRNA sequences should be designed to directly translate into proteins upon entering host cells. In contrast, for negative-sense ssRNA viruses, the saRNA design needs to account for the complementary nature of the viral mRNA, ensuring an intermediate step of transcription into positive-sense RNA before translation occurs. The challenge lies in precisely mimicking the viral genomic elements while maintaining the inherent safety and stability associated with saRNA technology [96,99,100]. Strategies may involve integrating coding sequences for essential viral proteins and utilizing subgenomic promoters to drive the expression of genes of interest. Careful consideration of the unique replication strategies of each virus type is crucial for achieving effective and controlled gene expression within the saRNA platform.

Expanding on the insights garnered from the current study, it is imperative to recognize that within the domain of oncolytic virotherapy, a myriad of viruses, each harboring unique oncolytic potential, can be explored beyond the exemplified Zika virus (ZIKV). In addition to ZIKV, other oncolytic viruses, including adenoviruses, herpesviruses, and measles viruses, exhibit distinct genomic attributes that can be strategically employed in the design of protein compositions [24,30]. This strategic approach seeks to optimize oncolytic potential while simultaneously mitigating the inherent risks associated with live virus applications. This inclusive exploration of different oncolytic viruses, coupled with the utilization of saRNA technology, enriches the oncolytic virotherapy toolkit. Not only does it broaden the spectrum of therapeutic options, it also underscores the potential for a nuanced and personalized approach in the ongoing pursuit of effective cancer treatments [21,24]. Continued investigation of various oncolytic viruses, augmented by the capabilities of saRNA technology, holds the promise of advancing precision medicine in cancer therapeutics.

Early clinical studies with oncolytic viruses typically involve direct intratumoral injection [36]. However, platforms based on saRNA technology offer a versatile application approach. They can be administered through direct intratumoral injection or adapted to address specific challenges, such as the formidable barrier posed by the blood–brain barrier (BBB) in combatting brain tumors like GBM [101,102,103]. Overcoming the selective permeability of the BBB is crucial for effective drug delivery to the central nervous system (CNS). In preclinical studies, nanometric drug carriers have emerged as efficient therapeutic modalities. For instance, psychostimulant drugs like amphetamine and methylated amphetamine (METH) have demonstrated BBB penetration. In a groundbreaking approach, the researchers designed, synthesized, and formulated three distinct β-amphetaminylated cationic lipid nanoparticles. These nanoparticles proved to be non-toxic and capable of crossing the BBB, potentially through active transcytosis [104]. The ability to tailor these lipid nanoparticles, with the hydrophilic-hydrophobic balance influencing BBB penetration, holds promise for diverse therapeutic applications, including saRNA platform’s.

## 4. Conclusions

The exploration of oncolytic virotherapy and its convergence with self-amplifying RNA technology reveal a pivotal juncture in the advancement of cancer therapeutics. Synergistic fusion of these cutting-edge methods harnesses the intrinsic potential of viruses as targeted therapeutic agents while leveraging their multifunctional capabilities to drive a refined immune response. This innovative and as yet unexplored approach, rooted in the use of viruses as potent therapeutic agents, holds great promise in the battle against these aggressive brain tumors. Oncolytic virotherapy capitalizes on the selective infectivity and replication of viruses in cancer cells, destroying them while sparing healthy tissues. Engineered virotherapies amplify this impact through enhancements in tumor selectivity, replication efficiency, and immune activation, launching a comprehensive assault on the tumor microenvironment. This, coupled with the potential release of tumor-specific antigens, fuels robust antitumor immune responses. A spectrum of viruses, including adenoviruses, herpesviruses, and measles viruses, has demonstrated promising outcomes in preclinical and clinical settings, showcasing their potential to shape the landscape of cancer treatment strategies. Among the array of viruses that have yielded promising outcomes, the Zika virus’s oncolytic potential against glioblastomas offers a novel and targeted avenue. The Zika virus, with its unique ability to preferentially infect and eliminate glioblastoma stem cells, holds great promise for circumventing the challenges associated with conventional treatments. This innovative approach aligns the fields of virology and oncology, exemplifying viruses as potent allies in the pursuit of enhanced, targeted, and personalized cancer therapies. As we explore the integration of oncolytic virotherapy with saRNA technology, the potential for customized, adaptable, and curative immune responses against glioblastomas becomes increasingly evident. Similarly, the distinctive capacity of saRNAs for self-replication and multifunctional encoding augments their potential as a transformative genetic therapy, effectively amplifying protein expression while reducing potential off-target effects. This visionary integrative approach allows the simultaneous targeting of multiple facets of tumor biology and immune-response modulation. The convergence of oncolytic virotherapy and saRNA technology using precision genetics is a monumental advance, paving the way for a highly adaptable, versatile, and potentially curative immune response against cancer and could ultimately revolutionize treatment for glioblastomas.

## Figures and Tables

**Table 1:** The twenty-eight clinical studies employing oncolytic viruses for the theatment of brain neoplasms, including Glioblastoma multiforme.

NCT Number	Study Title	Virus	Study Status	Phases
NCT00028158	Safety and effectiveness study of G207, a tumor-killing virus, in patients with recurrent brain cancer	Herpes simplex virus type 1	Completed	Phase 1/2
NCT00528684	Safety and efficacy study of REOLYSIN^®^ in the treatment of recurrent malignant gliomas	Reovirus	Completed	Phase 1
NCT01174537	New Castle disease virus in glioblastoma multiforme (GBM), sarcoma and neuroblastoma	New Castle virus	Withdrawn	Phase 1/2
NCT01301430	Parvovirus H-1 (ParvOryx) in patients with progressive primary or recurrent GBM.	Parvovirus H-1	Completed	Phase 1/2
NCT01491893	PVSRIPO for recurrent GBM	Poliovirus serotype 1 + Human Rhinovirus type 2	Completed	Phase 1
NCT01582516	Safety study of replication-competent adenovirus (Delta-24-rgd) in patients with recurrent glioblastoma	Adenovirus Delta24-RGD	Completed	Phase 1/2
NCT01956734	Virus DNX2401 and temozolomide in recurrent glioblastoma	Adenovirus Delta24-RGD	Completed	Phase 1
NCT02031965	Oncolytic HSV-1716 in treating younger patients with refractory or recurrent high-grade glioma that can be removed by surgery	Herpes simplex virus	Terminated	Phase 1
NCT02062827	Genetically engineered HSV-1 phase 1 study for the treatment of recurrent malignant glioma	Herpes simplex virus type 1	Active_Not_Recruiting	Phase 1
NCT02197169	DNX-2401 with Interferon Gamma (IFN-γ) for recurrent glioblastoma or gliosarcoma brain tumors	Adenovirus Delta24-RGD	Completed	Phase 1
NCT02457845	HSV G207 alone or with a single radiation dose in children with progressive or recurrent supratentorial brain tumors	Herpes simplex virus type 1	Active_Not_Recruiting	Phase 1
NCT02798406	Combination adenovirus + pembrolizumab to trigger immune virus effects	Adenovirus Delta24-RGD	Completed	Phase 2
NCT03043391	Phase 1b study PVSRIPO for recurrent malignant glioma in children	Poliovirus	Unknown	Phase 1
NCT03072134	Neural stem cell-based virotherapy of newly diagnosed malignant glioma	Adenovirus	Completed	Phase 1
NCT03152318	a study of the treatment of recurrent malignant glioma with rQNestin34.5v.2	Herpes simplex virus type 1	Recruiting	Phase 1
NCT03178032	Oncolytic adenovirus, DNX-2401, for naive diffuse intrinsic pontine gliomas	Adenovirus Delta24-RGD	Completed	Phase 1
NCT03294486	Safety and efficacy of the ONCOlytic VIRus armed for local chemotherapy, TG6002/5-FC, in recurrent Glioblastoma patients	Vaccinia virus	Unknown	Phase 1/2
NCT03657576	Trial of C134 in patients with recurrent GBM	Herpes simplex virus type 1	Recruiting	Phase 1
NCT03714334	DNX-2440 Oncolytic Adenovirus for recurrent glioblastoma	Adenovirus	Terminated	Phase 1
NCT03896568	MSC-DNX-2401 in treating patients with recurrent high-grade glioma	Adenovirus Delta24-RGD	Recruiting	Phase 1
NCT03911388	HSV G207 in children with recurrent or refractory cerebellar brain tumors	Herpes simplex virus type 1	Recruiting	Phase 1
NCT04482933	HSV G207 with a single radiation dose in children with recurrent high-grade glioma	Herpes simplex virus type 1	Not_Yet_Recruiting	Phase 2
NCT04758533	Clinical trial to assess the safety and efficacy of AloCELYVIR with newly diagnosed diffuse intrinsic pontine glioma in combination with radiotherapy or Medulloblastoma in monotherapy	Adenovirus	Recruiting	Phase 1/2
NCT05084430	Study of Pembrolizumab and M032 (NSC 733972)	Herpes simplex virus type 1	Recruiting	Phase 1/2
NCT05139056	Multiple doses of neural stem cell virotherapy (NSC-CRAd-S-pk7) for the treatment of recurrent high-grade gliomas	Adenovirus	Recruiting	Phase 1
NCT05235074	OH2 Oncolytic viral therapy in central nervous system tumors	Herpes simplex virus type 2	Recruiting	Phase 1/2
NCT05717699	Oncolytic virus Ad-TD-nsIL12 for progressive pediatric diffuse intrinsic pontine glioma	Adenovirus	Recruiting	Phase 1
NCT05717712	Oncolytic virus Ad-TD-nsIL12 for primary pediatric diffuse intrinsic pontine glioma	Adenovirus	Recruiting	Phase 1

**Table 2:** Examples of viral proteins and their oncolytic effects.

Protein	Oncolytic Effect	Virus	References
*NS1*	Cell death was to be induced by apoptosis and dependent on caspase-9-driven caspase-3 activation.	Parvovirus	[81,82]
*Rep78*	By protecting shielding p53 from ubiquitin-mediated degradation by adenovirus, the, it restores p53’s function of p53 as a cell cycle blocking agent is restored in the presence of Rep78.	Adeno-Associated Viruses (AAV)	[83]
*Rep6/U94*	Negatively modulating DDR (DNA Damage Response)-related genes, cholesterol biosynthesis, and cell cycle regulation, while inducing apoptotic cell death through activation of the intrinsic apoptotic pathway, thereby inhibiting tumor progression and metastasis in both in vivo and in vitro models.	Human Herpesvirus Type 6 (HHV-6)	[84]
*Apoptin*	Initiates caspase-mediated cell death through the intrinsic apoptotic pathway, operating independently of p53, yet necessitating pro-apoptotic transcriptionally active p73 isoforms belonging to the p53 family.	Chicken Anemia Virus (CAV)	[85,86,87,88,89]
*p17*	Via its nucleocytoplasmic shuttling mechanism and interaction with cellular proteins such as hnRNP A1 and Tpr, it prompts a deceleration in cell growth by activating CDK inhibitors, subsequently suppressing the PI3K/Akt/mTOR and ERK signaling pathways.	Avian Reovirus (ARV)	[90]
*p10.8*	Capacity to initiate apoptosis in DF-1 and VERO cells.	Muscovy Duck Reovirus (MDRV)	[91]
*F Protein*	Induce potential oncolytic effects, possibly through the inhibition of mTORC1. This suggests its participation in modulating autophagy.	Newcastle Disease Virus (NDV)	[92]
*E1/E2*	The structural envelope proteins E1 and E2 of SINV demonstrate cytotoxicity, wherein E1 exhibits more pronounced cytotoxic effects than E2 in human neuroblastoma cell lines (NB69, NGP, and RT-BM-1).	Sindbis virus (SINV)	[93,94]
*N protein*	Cell death due to the generation of reactive oxygen species (ROS) and the activation of Caspase 3.	Measles	[95]
*NS5*	Suppressed proliferation, migration, and invasion of cells.	Zika virus	[46]

## Data Availability

Not applicable.

## Supplementary Materials

The following supporting information can be downloaded at: https://www.mdpi.com/article/10.3390/vaccines12010061/s1, Table S1: A total of 196 studies are registered on the clinicaltrials platform involving oncolytic viruses.
