# Supplementary material for: Breaking Barriers: A Future Perspective on Glioblastoma Therapy with mRNA-Based Immunotherapies and Oncolytic Viruses
Source: Vaccines (Basel). 2024 Jan 8;12(1):61. doi: 10.3390/vaccines12010061 (PMC10818651; doi:10.3390/vaccines12010061)
Supplement: Supplementary file 1 [file vaccines-12-00061-s001.zip › vaccines-2773663-supplementary.pdf]

NCT Number	Study Title	Study Status	Conditions	Interventions	Phases	Study Type
NCT00028158	Safety and Effectiveness Study of G207, a Tumor-Killing Virus, in Patients With Recurrent Brain Cancer	COMPLETED	Glioma|Astrocytoma|Glioblastoma	DRUG: G207, an oncolytic virus	PHASE1|PHASE2	INTERVENTIONAL
NCT00092222	Virotherapy and Natural History Study of KHSV-Associated Multicentric Castleman s Disease With Correlates of Disease Activity	ACTIVE_NOT_RECRUITING	Lymphoproliferative Disorder|HHV-8|Malignancy|HIV	DRUG: Etoposide|DRUG: Interferon-alpha|DRUG: Rituximab|DRUG: Zidovudine|DRUG: Liposomal Doxorubicin|DRUG: Bortezomib|DRUG: Valganciclovir|DRUG: Doxorubicin|DRUG: Vincristine|DRUG: Cyclophosphamide|DRUG: Filgrastim (G-CSF)|DRUG: Prednisone|DRUG: Sirolimus|OTHER: Observation Only	PHASE2	INTERVENTIONAL
NCT00289016	A Study of Talimogene Laherparepvec in Stage IIIc and Stage IV Malignant Melanoma	COMPLETED	Melanoma	DRUG: Talimogene Laherparepvec	PHASE2	INTERVENTIONAL
NCT00314925	Safety Study of Seneca Valley Virus in Patients With Solid Tumors With Neuroendocrine Features	UNKNOWN	Carcinoid|Neuroendocrine	DRUG: Seneca Valley Virus (biological agent)	PHASE1	INTERVENTIONAL
NCT00348842	Newcastle Disease Virus (NDV) for Cancer Patients Resistant to Conventional Anti-cancer Modalities	WITHDRAWN	Metastatic Cancer|Newcastle Disease Virus (NDV)	PROCEDURE: Newcastle Virus	PHASE2	INTERVENTIONAL
NCT00408590	Recombinant Measles Virus Vaccine Therapy and Oncolytic Virus Therapy in Treating Patients With Progressive, Recurrent, or Refractory Ovarian Epithelial Cancer or Primary Peritoneal Cancer	COMPLETED	Ovarian Cancer|Primary Peritoneal Cavity Cancer	BIOLOGICAL: carcinoembryonic antigen-expressing measles virus|BIOLOGICAL: oncolytic measles virus encoding thyroidal sodium iodide symporter|GENETIC: reverse transcriptase-polymerase chain reaction|OTHER: laboratory biomarker analysis	PHASE1	INTERVENTIONAL
NCT00429312	A Study of Recombinant Vaccinia Virus to Treat Malignant Melanoma	COMPLETED	Melanoma	BIOLOGICAL: JX-594	PHASE1|PHASE2	INTERVENTIONAL
NCT00438009	A Safety Study of Two Intratumoural Doses of Coxsackievirus Type A21 in Melanoma Patients (PSX-X03)	COMPLETED	Stage IV Melanoma	DRUG: Coxsackievirus A21	PHASE1	INTERVENTIONAL
NCT00450814	Vaccine Therapy With or Without Cyclophosphamide in Treating Patients With Recurrent or Refractory Multiple Myeloma	COMPLETED	Recurrent Plasma Cell Myeloma|Refractory Plasma Cell Myeloma	DRUG: Cyclophosphamide|OTHER: Laboratory Biomarker Analysis|BIOLOGICAL: Oncolytic Measles Virus Encoding Thyroidal Sodium Iodide Symporter|OTHER: Pharmacological Study	PHASE1|PHASE2	INTERVENTIONAL
NCT00503295	Safety and Efficacy Study of REOLYSIN® in the Treatment of Bone and Soft Tissue Sarcomas Metastatic to the Lung	COMPLETED	Osteosarcoma|Ewing Sarcoma Family Tumors|Malignant Fibrous Histiocytoma|Sarcoma, Synovial|Fibrosarcoma|Leiomyosarcoma	BIOLOGICAL: REOLYSIN®	PHASE2	INTERVENTIONAL
NCT00528684	Safety and Efficacy Study of REOLYSIN® in the Treatment of Recurrent Malignant Gliomas	COMPLETED	Malignant Glioma	BIOLOGICAL: REOLYSIN®	PHASE1	INTERVENTIONAL
NCT00554372	A Study of Recombinant Vaccinia Virus to Treat Unresectable Primary Hepatocellular Carcinoma	COMPLETED	Carcinoma, Hepatocellular	GENETIC: JX-594: Recombinant vaccinia virus (TK-deletion plus GM-CSF)	PHASE2	INTERVENTIONAL
NCT00625456	Safety Study of Recombinant Vaccinia Virus to Treat Refractory Solid Tumors	COMPLETED	Melanoma|Lung Cancer|Renal Cell Carcinoma|Squamous Cell Carcinoma of the Head and Neck	DRUG: Recombinant Vaccinia GM-CSF; RAC VAC GM-CSF (JX-594)	PHASE1	INTERVENTIONAL
NCT00629759	A Study of Recombinant Vaccinia Virus to Evaluate the Safety and Efficacy of a Transdermal Injection Within the Tumor of Patients With Primary or Metastatic Hepatic Carcinoma	COMPLETED	Neoplasms, Liver	GENETIC: JX-594: Recombinant vaccinia virus (TK-deletion plus GM-CSF)	PHASE1	INTERVENTIONAL
NCT00794131	Safety Study of GL-ONC1, an Oncolytic Virus, in Patients With Advanced Solid Tumors	COMPLETED	Advanced Cancers (Solid Tumors)	BIOLOGICAL: GL-ONC1	PHASE1	INTERVENTIONAL
NCT00832559	A Study of the Intratumoural Administration of CAVATAK to Head and Neck Cancer Patients (VLA-X06)	TERMINATED	Head and Neck Cancer	BIOLOGICAL: CVA21	PHASE1	INTERVENTIONAL
NCT00931931	HSV1716 in Patients With Non-Central Nervous System (Non-CNS) Solid Tumors	COMPLETED	Rhabdomyosarcoma|Osteosarcoma|Ewing Sarcoma|Soft Tissue Sarcoma|Neuroblastoma|Wilms Tumor|Malignant Peripheral Nerve Sheath Tumor|Clival Chordoma|Non-CNS Solid Tumors	BIOLOGICAL: HSV1716	PHASE1	INTERVENTIONAL
NCT01017185	Study of HF10 in Patients With Refractory Head and Neck Cancer or Solid Tumors With Cutaneous and/or Superficial Lesions	COMPLETED	Refractory Head and Neck Cancer|Squamous Cell Carcinoma, Skin|Carcinoma of the Breast|Malignant Melanoma	DRUG: HF10	PHASE1	INTERVENTIONAL
NCT01169584	Safety Study of Recombinant Vaccinia Virus to Treat Refractory Solid Tumors in Pediatric Patients	COMPLETED	Neuroblastoma|Rhabdomyosarcoma|Lymphoma|Wilm's Tumor|Ewing's Sarcoma	DRUG: Recombinant Vaccinia GM-CSF; RAC VAC GM-CSF (JX-594)	PHASE1	INTERVENTIONAL
NCT01174537	New Castle Disease Virus (NDV) in Glioblastoma Multiforme (GBM), Sarcoma and Neuroblastoma	WITHDRAWN	Glioblastoma|Sarcoma|Neuroblastoma	BIOLOGICAL: New Castle Disease Virus	PHASE1|PHASE2	INTERVENTIONAL
NCT01227551	A Study of Intratumoral CAVATAK™ in Patients With Stage IIIc and Stage IV Malignant Melanoma (VLA-007 CALM)	COMPLETED	Malignant Melanoma	BIOLOGICAL: Coxsackievirus A21 (CVA21)	PHASE2	INTERVENTIONAL
NCT01274624	Study of REOLYSIN® in Combination With FOLFIRI and Bevacizumab in FOLFIRI Naive Patients With KRAS Mutant Metastatic Colorectal Cancer	COMPLETED	KRAS Mutant Metastatic Colorectal Cancer	BIOLOGICAL: REOLYSIN®|DRUG: Irinotecan|DRUG: Leucovorin|DRUG: Fluorouracil (5-FU)|DRUG: Bevacizumab	PHASE1	INTERVENTIONAL
NCT01301430	Parvovirus H-1 (ParvOryx) in Patients With Progressive Primary or Recurrent Glioblastoma Multiforme.	COMPLETED	Glioblastoma Multiforme	DRUG: H-1PV	PHASE1|PHASE2	INTERVENTIONAL
NCT01387555	A Phase 2b Study of Modified Vaccinia Virus to Treat Patients Advanced Liver Cancer Who Failed Sorafenib	COMPLETED	Hepatocellular Carcinoma|Liver Cancer|HCC	BIOLOGICAL: JX-594 recombinant vaccina GM-CSF|OTHER: Best Supportive Care	PHASE2	INTERVENTIONAL
NCT01394939	Recombinant Vaccinia Virus Administered Intravenously in Patients With Metastatic, Refractory Colorectal Carcinoma	COMPLETED	Colorectal Carcinoma|CRC	BIOLOGICAL: JX-594|DRUG: Irinotecan	PHASE1|PHASE2	INTERVENTIONAL
NCT01437280	GOAT; Phase I Open Label Study of CGTG-102, a GM-CSF Encoding Oncolytic Adenovirus, for Advanced Cancers	WITHDRAWN	Tumors|Solid Tumors	BIOLOGICAL: CGTG-102	PHASE1	INTERVENTIONAL
NCT01438112	Efficacy Study of Recombinant Adenovirus for Non Muscle Invasive Bladder Cancer	TERMINATED	Transitional Cell Carcinoma|Bladder Cancer|Carcinoma in Situ|Carcinoma in Situ Concurrent With Papillary Tumors	BIOLOGICAL: CG0070 Adenovirus Vector|OTHER: Control Arm: Quadruple Choice	PHASE2|PHASE3	INTERVENTIONAL
NCT01443260	A Study of GL-ONC1, an Oncolytic Vaccinia Virus, in Patients With Advanced Peritoneal Carcinomatosis	COMPLETED	Peritoneal Carcinomatosis	BIOLOGICAL: GL-ONC1	PHASE1|PHASE2	INTERVENTIONAL
NCT01491893	PVSRIPO for Recurrent Glioblastoma (GBM)	COMPLETED	GBM|Glioblastoma|Glioma|Malignant Glioma	BIOLOGICAL: Recombinant nonpathogenic polio-rhinovirus chimera (PVSRIPO)	PHASE1	INTERVENTIONAL
NCT01503177	Intrapleural Measles Virus Therapy in Patients With Malignant Pleural Mesothelioma	COMPLETED	Recurrent Malignant Mesothelioma|Stage IA Malignant Mesothelioma|Stage IB Malignant Mesothelioma|Stage II Malignant Mesothelioma|Stage III Malignant Mesothelioma|Stage IV Malignant Mesothelioma	BIOLOGICAL: oncolytic measles virus encoding thyroidal sodium iodide symporter|OTHER: laboratory biomarker analysis|PROCEDURE: single photon emission computed tomography|PROCEDURE: computed tomography	PHASE1	INTERVENTIONAL
NCT01582516	Safety Study of Replication-competent Adenovirus (Delta-24-rgd) in Patients With Recurrent Glioblastoma	COMPLETED	Brain Tumor|Recurring Glioblastoma	BIOLOGICAL: delta-24-RGD adenovirus	PHASE1|PHASE2	INTERVENTIONAL
NCT01584284	Safety Study of Attenuated Vaccinia Virus (GL-ONC1)With Combination Therapy in Head & Neck Cancer	COMPLETED	Cancer of Head and Neck	BIOLOGICAL: GL-ONC1	PHASE1	INTERVENTIONAL
NCT01598129	ONCOS-102 (Previously CGTG-102) for Therapy of Advanced Cancers	COMPLETED	Malignant Solid Tumour	GENETIC: ONCOS-102	PHASE1	INTERVENTIONAL
NCT01721018	Intrapleural Administration of HSV1716 to Treat Patients With Malignant Pleural Mesothelioma.	COMPLETED	Malignant Pleural Mesothelioma	BIOLOGICAL: HSV1716 Intra-pleural delivery	PHASE1|PHASE2	INTERVENTIONAL
NCT01846091	Viral Therapy In Treating Patients With Recurrent or Metastatic Squamous Cell Carcinoma of the Head and Neck Cancer or Metastatic Breast Cancer	COMPLETED	Estrogen Receptor Negative|Estrogen Receptor Positive|Head and Neck Squamous Cell Carcinoma|HER2/Neu Negative|HER2/Neu Positive|Invasive Breast Carcinoma|Progesterone Receptor Negative|Progesterone Receptor Positive|Recurrent Head and Neck Carcinoma|Stage IV Breast Cancer|Triple-Negative Breast Carcinoma	OTHER: Laboratory Biomarker Analysis|BIOLOGICAL: Oncolytic Measles Virus Encoding Thyroidal Sodium Iodide Symporter	PHASE1	INTERVENTIONAL
NCT01864759	Phase I Endovenous Administration of Oncolytic Adenovirus ICOVIR-5 in Patients With Advanced or Metastatic Melanoma	COMPLETED	Locally Advanced or Metastatic Melanoma	BIOLOGICAL: ICOVIR-5	PHASE1	INTERVENTIONAL
NCT01956734	Virus DNX2401 and Temozolomide in Recurrent Glioblastoma	COMPLETED	Glioblastoma Multiforme|Recurrent Tumor	PROCEDURE: DNX2401 and Temozolomide	PHASE1	INTERVENTIONAL
NCT02017678	Phase 2 Study of JX-594 in Patients With Peritoneal Carcinomatosis of Ovarian Cancer Origin	WITHDRAWN	Ovarian Cancer	BIOLOGICAL: JX-594	PHASE2	INTERVENTIONAL
NCT02028117	Phase I / Dose Expansion Study of Enadenotucirev in Ovarian Cancer Patients	COMPLETED	Recurrent Platinum Resistant Ovarian Cancer	BIOLOGICAL: Enadenotucirev	PHASE1	INTERVENTIONAL
NCT02028442	Phase I / II Study of Enadenotucirev by Sub-acute Fractionated IV Dosing in Cancer Patients	COMPLETED	Solid Tumours of Epithelial Origin|Metastatic Colorectal Cancer|Metastatic Bladder Cancer	BIOLOGICAL: Enadenotucirev	PHASE1|PHASE2	INTERVENTIONAL
NCT02031965	Oncolytic HSV-1716 in Treating Younger Patients With Refractory or Recurrent High Grade Glioma That Can Be Removed By Surgery	TERMINATED	Recurrent Childhood Anaplastic Astrocytoma|Recurrent Childhood Anaplastic Oligoastrocytoma|Recurrent Childhood Anaplastic Oligodendroglioma|Recurrent Childhood Giant Cell Glioblastoma|Recurrent Childhood Glioblastoma|Recurrent Childhood Gliomatosis Cerebri|Recurrent Childhood Gliosarcoma	BIOLOGICAL: oncolytic HSV-1716|DRUG: dexamethasone|PROCEDURE: therapeutic conventional surgery|OTHER: laboratory biomarker analysis	PHASE1	INTERVENTIONAL
NCT02045589	A Phase I Dose Escalation Study of Intratumoral VCN-01 Injections With Gemcitabine and Abraxane® in Patients With Advanced Pancreatic Cancer	COMPLETED	Pancreatic Adenocarcinoma|Metastatic Pancreatic Adenocarcinoma	GENETIC: VCN-01|DRUG: Gemcitabine|DRUG: Abraxane®	PHASE1	INTERVENTIONAL

NCT02045602	Phase I Dose Escalation Study of Intravenous VCN-01 With or Without Gemcitabine and Abraxane® in Patients With Advanced Solid Tumors	COMPLETED	Locally Advanced Solid Tumors|Metastatic Solid Tumors|Pancreatic Adenocarcinoma	GENETIC: VCN-01|DRUG: Gemcitabine|DRUG: Abraxane®	PHASE1	INTERVENTIONAL
NCT02053220	Mechanism of Action Trial of ColoAd1	COMPLETED	Resectable Colon Cancer|Resectable Non-small Cell Lung Cancer|Resectable Bladder Cancer|Resectable Renal Cell Carcinoma	BIOLOGICAL: Colo-Ad1	PHASE1	INTERVENTIONAL
NCT02062827	Genetically Engineered HSV-1 Phase 1 Study for the Treatment of Recurrent Malignant Glioma	ACTIVE_NOT_RECRUITING	Recurrent Glioblastoma Multiforme|Progressive Glioblastoma Multiforme|Anaplastic Astrocytoma or Gliosarcoma	BIOLOGICAL: M032 (NSC 733972)	PHASE1	INTERVENTIONAL
NCT02068794	MV-NIS Infected Mesenchymal Stem Cells in Treating Patients With Recurrent Ovarian, Primary Peritoneal or Fallopian Tube Cancer	RECRUITING	Fallopian Tube Clear Cell Adenocarcinoma|Fallopian Tube Endometrioid Adenocarcinoma|Fallopian Tube Mucinous Adenocarcinoma|Fallopian Tube Serous Adenocarcinoma|Fallopian Tube Transitional Cell Carcinoma|Fallopian Tube Undifferentiated Carcinoma|Malignant Ovarian Brenner Tumor|Ovarian Clear Cell Adenocarcinoma|Ovarian Endometrioid Adenocarcinoma|Ovarian Mucinous Adenocarcinoma|Ovarian Seromucinous Carcinoma|Ovarian Serous Adenocarcinoma|Ovarian Transitional Cell Carcinoma|Ovarian Undifferentiated Carcinoma|Primary Peritoneal Serous Adenocarcinoma|Recurrent Fallopian Tube Carcinoma|Recurrent Ovarian Carcinoma|Recurrent Primary Peritoneal Carcinoma	OTHER: Laboratory Biomarker Analysis|PROCEDURE: Mesenchymal Stem Cell Transplantation|BIOLOGICAL: Oncolytic Measles Virus Encoding Thyroidal Sodium Iodide Symporter	PHASE1|PHASE2	INTERVENTIONAL
NCT02143804	Safety and Efficacy of CG0070 Oncolytic Virus Regimen in Patients With High Grade Non-Muscle Invasive Bladder Cancer	WITHDRAWN	Bladder Cancer|High Grade|Non Muscle Invasive	BIOLOGICAL: oncolytic adenovirus expressing GMCSF	PHASE2	INTERVENTIONAL
NCT02192775	UARK 2014-21 A Phase II Trial of Oncolytic Virotherapy by Systemic Administration of Edmonston Strain of Measles Virus	COMPLETED	Multiple Myeloma	DRUG: MV-NIS	PHASE2	INTERVENTIONAL
NCT02197169	DNX-2401 With Interferon Gamma (IFN-γ) for Recurrent Glioblastoma or Gliosarcoma Brain Tumors	COMPLETED	Glioblastoma or Gliosarcoma	DRUG: Single intratumoral injection of DNX-2401|DRUG: Interferon-gamma	PHASE1	INTERVENTIONAL
NCT02272855	A Study of Combination Treatment With HF10 and Ipilimumab in Patients With Unresectable or Metastatic Melanoma	COMPLETED	Malignant Melanoma	BIOLOGICAL: HF10 plus Ipilimumab	PHASE2	INTERVENTIONAL
NCT02285816	MG1 Maraba/MAGE-A3, With and Without Adenovirus Vaccine With Transgenic MAGE-A3 Insertion in Incurable MAGE-A3-Expressing Solid Tumours	ACTIVE_NOT_RECRUITING	Advanced/Metastatic Solid Tumours	BIOLOGICAL: MG1MA3|BIOLOGICAL: AdMA3	PHASE1|PHASE2	INTERVENTIONAL
NCT02364713	MV-NIS or Investigator's Choice Chemotherapy in Treating Patients With Ovarian, Fallopian, or Peritoneal Cancer	ACTIVE_NOT_RECRUITING	Fallopian Tube Carcinosarcoma|Fallopian Tube Clear Cell Adenocarcinoma|Fallopian Tube Endometrioid Adenocarcinoma|Fallopian Tube Mucinous Adenocarcinoma|Fallopian Tube Serous Adenocarcinoma|Fallopian Tube Transitional Cell Carcinoma|Fallopian Tube Undifferentiated Carcinoma|Ovarian Carcinosarcoma|Ovarian Clear Cell Adenocarcinoma|Ovarian Endometrioid Adenocarcinoma|Ovarian Mucinous Adenocarcinoma|Ovarian Seromucinous Carcinoma|Ovarian Serous Adenocarcinoma|Ovarian Transitional Cell Carcinoma|Ovarian Undifferentiated Carcinoma|Platinum-Resistant Fallopian Tube Carcinoma|Platinum-Resistant Ovarian Carcinoma|Platinum-Resistant Primary Peritoneal Carcinoma|Primary Peritoneal Carcinosarcoma|Primary Peritoneal Clear Cell Adenocarcinoma|Primary Peritoneal Endometrioid Adenocarcinoma|Primary Peritoneal Serous Adenocarcinoma|Primary Peritoneal Transitional Cell Carcinoma|Primary Peritoneal Undifferentiated Carcinoma|Recurrent Fallopian Tube Carcinoma|Recurrent Ovarian Carcinoma|Recurrent Primary Peritoneal Carcinoma	BIOLOGICAL: Bevacizumab|DRUG: Gemcitabine Hydrochloride|OTHER: Laboratory Biomarker Analysis|BIOLOGICAL: Oncolytic Measles Virus Encoding Thyroidal Sodium Iodide Symporter|DRUG: Paclitaxel|DRUG: Pegylated Liposomal Doxorubicin Hydrochloride|OTHER: Quality-of-Life Assessment|DRUG: Topotecan Hydrochloride	PHASE2	INTERVENTIONAL
NCT02365818	Safety and Efficacy of CG0070 Oncolytic Virus Regimen for High Grade NMIBC After BCG Failure	COMPLETED	Bladder Cancer	BIOLOGICAL: CG0070	PHASE2	INTERVENTIONAL
NCT02428036	A Study of TBI-1401(HF10) in Patients With Solid Tumors With Superficial Lesions	COMPLETED	Solid Tumor	BIOLOGICAL: TBI-1401(HF10)|BIOLOGICAL: TBI-1401(HF10)	PHASE1	INTERVENTIONAL
NCT02457845	HSV G207 Alone or With a Single Radiation Dose in Children With Progressive or Recurrent Supratentorial Brain Tumors	ACTIVE_NOT_RECRUITING	Supratentorial Neoplasms, Malignant|Malignant Glioma|Glioblastoma|Anaplastic Astrocytoma|PNET|Cerebral Primitive Neuroectodermal Tumor|Embryonal Tumor	BIOLOGICAL: G207	PHASE1	INTERVENTIONAL
NCT02562755	Hepatocellular Carcinoma Study Comparing Vaccinia Virus Based Immunotherapy Plus Sorafenib vs Sorafenib Alone	COMPLETED	Hepatocellular Carcinoma (HCC)	BIOLOGICAL: Pexastimogene Devacirepvec (Pexa Vec)|DRUG: Sorafenib	PHASE3	INTERVENTIONAL
NCT02620423	Study of Pembrolizumab With REOLYSIN® and Chemotherapy in Patients With Advanced Pancreatic Adenocarcinoma	COMPLETED	Pancreatic Adenocarcinoma	BIOLOGICAL: REOLYSIN®|DRUG: Chemotherapy|DRUG: Gemcitabine|DRUG: Irinotecan|DRUG: Leucovorin|DRUG: 5-fluorouracil|DRUG: Pembrolizumab	PHASE1	INTERVENTIONAL
NCT02700230	Vaccine Therapy in Treating Patients With Malignant Peripheral Nerve Sheath Tumor That is Recurrent or Cannot Be Removed by Surgery	RECRUITING	Metastatic Malignant Peripheral Nerve Sheath Tumor|Neurofibromatosis Type 1|Recurrent Malignant Peripheral Nerve Sheath Tumor	PROCEDURE: Computed Tomography|OTHER: Laboratory Biomarker Analysis|BIOLOGICAL: Oncolytic Measles Virus Encoding Thyroidal Sodium Iodide Symporter|OTHER: Quality-of-Life Assessment|PROCEDURE: Single Photon Emission Computed Tomography	PHASE1	INTERVENTIONAL
NCT02705196	LOAd703 Oncolytic Virus Therapy for Pancreatic Cancer	RECRUITING	Pancreatic Cancer	GENETIC: delolimogene mupadenorepvec|DRUG: gemcitabine|DRUG: nab-paclitaxel|BIOLOGICAL: atezolizumab	PHASE1|PHASE2	INTERVENTIONAL
NCT02714374	Safety and Effect of GL-ONC1 Administered IV Prior to Surgery to Patients With Solid Organ Cancers Undergoing Surgery	TERMINATED	Solid Organ Cancers	BIOLOGICAL: GL-ONC1	PHASE1	INTERVENTIONAL
NCT02723838	Study of Intratumoral REOLYSIN® in Combination With Gemcitabine and Cisplatin as Neoadjuvant Therapy in Muscle-invasive Transitional Cell Carcinoma of the Bladder	WITHDRAWN	Muscle-invasive Transitional Cell Carcinoma of the Bladder	BIOLOGICAL: REOLYSIN®|DRUG: Gemcitabine|DRUG: Cisplatin	PHASE1	INTERVENTIONAL
NCT02759588	GL-ONC1 Oncolytic Immunotherapy in Patients With Recurrent or Refractory Ovarian Cancer	COMPLETED	Ovarian Cancer|Peritoneal Carcinomatosis|Fallopian Tube Cancer	BIOLOGICAL: GL-ONC1 alone, or in combination with chemotherapy with or without bevacizumab	PHASE1|PHASE2	INTERVENTIONAL
NCT02779855	Talimogene Laherparepvec in Combination With Neoadjuvant Chemotherapy in Triple Negative Breast Cancer	ACTIVE_NOT_RECRUITING	Breast Cancer|Ductal Carcinoma|Invasive Breast Carcinoma|Invasive Ductal Breast Carcinoma	BIOLOGICAL: Talimogene laherparepvec|DRUG: Paclitaxel	PHASE1|PHASE2	INTERVENTIONAL
NCT02798406	Combination Adenovirus + Pembrolizumab to Trigger Immune Virus Effects	COMPLETED	Brain Cancer|Brain Neoplasm|Glioma|Glioblastoma|Gliosarcoma|Malignant Brain Tumor|Neoplasm, Neuroepithelial|Neuroectodermal Tumors|Neoplasm by Histologic Type|Neoplasm, Nerve Tissue|Nervous System Diseases	BIOLOGICAL: DNX-2401|BIOLOGICAL: pembrolizumab	PHASE2	INTERVENTIONAL
NCT02824965	Pembrolizumab + CVA21 in Advanced NSCLC	UNKNOWN	Non-Small Cell Lung Cancer	DRUG: Pembrolizumab|BIOLOGICAL: CVA21	PHASE1	INTERVENTIONAL
NCT02879760	Oncolytic MG1-MAGEA3 With Ad-MAGEA3 Vaccine in Combination With Pembrolizumab for Non-Small Cell Lung Cancer Patients	COMPLETED	Non-Small Cell Lung Cancer	BIOLOGICAL: Ad-MAGEA3|BIOLOGICAL: MG1-MAGEA3|BIOLOGICAL: Pembrolizumab	PHASE1|PHASE2	INTERVENTIONAL
NCT02977156	Immunization Strategy With Intra-tumoral Injections of Pexa-Vec With Ipilimumab in Metastatic / Advanced Solid Tumors.	COMPLETED	Metastatic Tumor|Advanced Tumor	BIOLOGICAL: Pexa-Vec|DRUG: Ipilimumab	PHASE1	INTERVENTIONAL
NCT03004183	SBRT and Oncolytic Virus Therapy Before Pembrolizumab for Metastatic TNBC and NSCLC	ACTIVE_NOT_RECRUITING	Metastatic Non-small Cell Lung Cancer|Metastatic Triple-negative Breast Cancer	BIOLOGICAL: ADV/HSV-tk|DRUG: Valacyclovir|RADIATION: SBRT|DRUG: Pembrolizumab	PHASE2	INTERVENTIONAL

NCT03017820	VSV-hIFNbeta-NIS With or Without Cyclophosphamide or Ipilimumab and Nivolumab in Treating Patients With Relapsed or Refractory Multiple Myeloma, Acute Myeloid Leukemia or Lymphoma	RECRUITING	B-Cell Non-Hodgkin Lymphoma|Histiocytic and Dendritic Cell Neoplasm|Myelodysplastic Syndrome|Previously Treated Myelodysplastic Syndrome|Recurrent Adult Acute Myeloid Leukemia|Recurrent Anaplastic Large Cell Lymphoma|Recurrent Angioimmunoblastic T-Cell Lymphoma|Recurrent Mycosis Fungoides|Recurrent Plasma Cell Myeloma|Recurrent Primary Cutaneous T-Cell Non-Hodgkin Lymphoma|Recurrent T-Cell Non-Hodgkin Lymphoma|Refractory Acute Myeloid Leukemia|Refractory Anaplastic Large Cell Lymphoma|Refractory Angioimmunoblastic T-Cell Lymphoma|Refractory Mycosis Fungoides|Refractory Peripheral T-Cell Lymphoma, Not Otherwise Specified|Refractory Plasma Cell Myeloma|Refractory Primary Cutaneous T-Cell Non-Hodgkin Lymphoma|Refractory T-Cell Non-Hodgkin Lymphoma	PROCEDURE: Biopsy|PROCEDURE: Biospecimen Collection|PROCEDURE: Bone Marrow Biopsy|PROCEDURE: Computed Tomography|DRUG: Cyclophosphamide|BIOLOGICAL: Ipilimumab|BIOLOGICAL: Nivolumab|PROCEDURE: Positron Emission Tomography|BIOLOGICAL: Recombinant Vesicular Stomatitis Virus-expressing Human Interferon Beta and Sodium-Iodide Symporter|PROCEDURE: Single Photon Emission Computed Tomography	PHASE1	INTERVENTIONAL
NCT03029871	Oncolytic Adenovirus-Mediated Gene Therapy for Lung Cancer	WITHDRAWN	Non-small Cell Lung Cancer Stage I	BIOLOGICAL: Ad5-yCD/mutTKSR39rep-ADP Adenovirus	PHASE1	INTERVENTIONAL
NCT03043391	Phase 1b Study PVSRIPO for Recurrent Malignant Glioma in Children	UNKNOWN	Malignant Glioma|Anaplastic Astrocytoma|Anaplastic Oligoastrocytoma|Anaplastic Oligodendroglioma|Glioblastoma|Gliosarcoma|Atypical Teratoid/Rhabdoid Tumor of Brain|Medulloblastoma|Ependymoma|Pleomorphic Xanthoastrocytoma of Brain|Embryonal Tumor of Brain	BIOLOGICAL: Polio/Rhinovirus Recombinant (PVSRIPO)	PHASE1	INTERVENTIONAL
NCT03072134	Neural Stem Cell Based Virotherapy of Newly Diagnosed Malignant Glioma	COMPLETED	Glioma|Anaplastic Astrocytoma|Anaplastic Oligodendroglioma|Anaplastic Oligoastrocytoma|Glioblastoma Multiforme|Astrocytoma, Grade III|Astrocytoma, Grade IV|Brain Cancer	BIOLOGICAL: Neural stem cells loaded with an oncolytic adenovirus	PHASE1	INTERVENTIONAL
NCT03120624	VSV-hIFNbeta-NIS With or Without Ruxolitinib Phosphate in Treating Patients With Stage IV or Recurrent Endometrial Cancer	ACTIVE_NOT_RECRUITING	Metastatic Endometrial Carcinoma|Recurrent Endometrial Adenocarcinoma|Recurrent Endometrial Carcinoma|Recurrent Endometrial Clear Cell Adenocarcinoma|Recurrent Endometrial Endometrioid Adenocarcinoma|Recurrent Endometrial Mixed Cell Adenocarcinoma|Recurrent Endometrial Serous Adenocarcinoma|Recurrent Endometrial Undifferentiated Carcinoma|Recurrent Uterine Corpus Carcinosarcoma|Stage IV Uterine Corpus Cancer AJCC v7|Stage IVA Uterine Corpus Cancer AJCC v7|Stage IVB Uterine Corpus Cancer AJCC v7	PROCEDURE: Biopsy|PROCEDURE: Computed Tomography|OTHER: Fluorine F 18 Tetrafluoroborate|OTHER: Laboratory Biomarker Analysis|OTHER: Pharmacological Study|PROCEDURE: Planar Imaging|PROCEDURE: Positron Emission Tomography|BIOLOGICAL: Recombinant Vesicular Stomatitis Virus-expressing Human Interferon Beta and Sodium-Iodide Symporter|DRUG: Ruxolitinib|DRUG: Ruxolitinib Phosphate|PROCEDURE: Single Photon Emission Computed Tomography|DRUG: Technetium Tc-99m Sodium Pertechnetate	PHASE1	INTERVENTIONAL
NCT03152318	A Study of the Treatment of Recurrent Malignant Glioma With rQNestin34.5v.2	RECRUITING	Malignant Glioma of Brain|Astrocytoma|Malignant Astrocytoma|Oligodendroglioma|Anaplastic Oligodendroglioma of Brain (Diagnosis)|Mixed Oligo-Astrocytoma|Ependymoma|Ganglioglioma|Pylocytic/Pylomyxoid Astrocytoma|Brain Tumor|Glioma|Brain Cancer|Glioblastoma|Glioblastoma Multiforme	DRUG: rQNestin|DRUG: Cyclophosphamide|PROCEDURE: Stereotactic biopsy	PHASE1	INTERVENTIONAL
NCT03153085	A Study of Combination With TBI-1401(HF10) and Ipilimumab in Japanese Patients With Unresectable or Metastatic Melanoma	COMPLETED	Melanoma Stage III|Melanoma Stage IV	BIOLOGICAL: TBI-1401(HF10)|DRUG: Ipilimumab	PHASE2	INTERVENTIONAL
NCT03171493	Trial of Intravesical Measles Virotherapy in Patients With Bladder Cancer Who Are Undergoing Radical Cystectomy	COMPLETED	Urothelial Carcinoma	BIOLOGICAL: MV-NIS	PHASE1	INTERVENTIONAL
NCT03178032	Oncolytic Adenovirus, DNX-2401, for Naive Diffuse Intrinsic Pontine Gliomas	COMPLETED	Brainstem Glioma|Neoadjuvant Therapy	BIOLOGICAL: DNX-2401	PHASE1	INTERVENTIONAL
NCT03190824	Evaluate Efficacy, Immunological Response of Intratumoral/Intralesional Oncolytic Virus (OBP-301) in Metastatic Melanoma	UNKNOWN	Melanoma Stage III|Melanoma Stage Iv	DRUG: OBP-301	PHASE2	INTERVENTIONAL
NCT03206073	A Phase I/II Study of Pexa-Vec Oncolytic Virus in Combination With Immune Checkpoint Inhibition in Refractory Colorectal Cancer	ACTIVE_NOT_RECRUITING	Colorectal Cancer|Colorectal Carcinoma|Colorectal Adenocarcinoma|Refractory Cancer|Colorectal Neoplasms	DRUG: Durvalumab|DRUG: Tremelimumab|BIOLOGICAL: Pexa-Vec|BIOLOGICAL: Pexa-Vec	PHASE1|PHASE2	INTERVENTIONAL
NCT03225989	Trial Investigating an Immunostimulatory Oncolytic Adenovirus for Cancer	ACTIVE_NOT_RECRUITING	Pancreatic Adenocarcinoma|Ovarian Cancer|Biliary Carcinoma|Colorectal Cancer	DRUG: LOAd703	PHASE1|PHASE2	INTERVENTIONAL
NCT03252808	Phase I Study of TBI-1401(HF10) Plus Chemotherapy in Patients With Unresectable Pancreatic Cancer.	ACTIVE_NOT_RECRUITING	Pancreatic Cancer Stage III|Pancreatic Cancer Stage IV	BIOLOGICAL: TBI-1401(HF10)|DRUG: Gemcitabine|DRUG: Nab-paclitaxel|DRUG: TS-1	PHASE1	INTERVENTIONAL
NCT03294083	A Study of Recombinant Vaccinia Virus in Combination With Cemiplimab for Renal Cell Carcinoma	ACTIVE_NOT_RECRUITING	Renal Cell Carcinoma	BIOLOGICAL: Pexastimogene Devacirepvec (Pexa-Vec)|BIOLOGICAL: Cemiplimab	PHASE1|PHASE2	INTERVENTIONAL
NCT03294486	Safety and Efficacy of the ONCOlytic VIRus Armed for Local Chemotherapy, TG6002/5-FC, in Recurrent Glioblastoma Patients	UNKNOWN	Glioblastoma|Brain Cancer	DRUG: Combination of TG6002 and 5-flucytosine (5-FC, Ancotil®)	PHASE1|PHASE2	INTERVENTIONAL
NCT03325517	Effect of Adenovirus E1A Oncogene on DNA Replication Dynamics	UNKNOWN	Adenovirus			OBSERVATIONAL
NCT03408587	CAVATAK® and Ipilimumab in Uveal Melanoma Metastatic to the Liver (VLA-024 CLEVER)	COMPLETED	Uveal Melanoma|Liver Metastases	BIOLOGICAL: CVA21|BIOLOGICAL: Ipilimumab	PHASE1	INTERVENTIONAL
NCT03529539	Expanded Access for PSV Personalized Oncolytic Viruses	NO_LONGER_AVAILABLE	Cancer	DRUG: PSV		XPANDED_ACCESS
NCT03605719	Dexamethasone, Carfilzomib, & Nivolumab With Pelareorep for Relapsed/Refractory Multiple Myeloma	ACTIVE_NOT_RECRUITING	Recurrent Plasma Cell Myeloma	DRUG: Carfilzomib|DRUG: Dexamethasone|BIOLOGICAL: Nivolumab|BIOLOGICAL: Pelareorep	PHASE1	INTERVENTIONAL
NCT03618953	This is a Trial of MG1-E6E7 With Ad-E6E7 and Atezolizumab in Patients With HPV Associated Cancers	TERMINATED	HPV-Associated Cancers	BIOLOGICAL: Ad-E6E7|BIOLOGICAL: MG1-E6E7|BIOLOGICAL: Atezolizumab	PHASE1	INTERVENTIONAL
NCT03647163	Ph I/II Trial of Systemic VSV-IFNβ-NIS in Combination With Checkpoint Inhibitor Therapy in Patients With Select Solid Tumors	RECRUITING	Solid Tumor|Non Small Cell Lung Cancer|Neuroendocrine Carcinoma	BIOLOGICAL: VSV-IFNβ-NIS|BIOLOGICAL: Pembrolizumab|BIOLOGICAL: ipilimumab + nivolumab	PHASE1|PHASE2	INTERVENTIONAL
NCT03657576	Trial of C134 in Patients With Recurrent GBM	RECRUITING	Glioblastoma Multiforme of Brain|Anaplastic Astrocytoma of Brain|Gliosarcoma of Brain	BIOLOGICAL: C134	PHASE1	INTERVENTIONAL
NCT03663712	Talimogene Laherparepvec for the Treatment of Peritoneal Surface Malignancies	COMPLETED	Stage IV Peritoneal Surface Dissemination From Gastrointestinal or Recurrent, Platinum-resistant Ovarian Cancer That Cannot be Completely Resected	BIOLOGICAL: Talimogene Laherparepvec	PHASE1	INTERVENTIONAL
NCT03714334	DNX-2440 Oncolytic Adenovirus for Recurrent Glioblastoma	TERMINATED	Glioblastoma|Glioblastoma, Adult	DRUG: DNX-2440 injection	PHASE1	INTERVENTIONAL
NCT03740256	Binary Oncolytic Adenovirus in Combination With HER2-Specific Autologous CAR VST, Advanced HER2 Positive Solid Tumors	RECRUITING	Bladder Cancer|Head and Neck Squamous Cell Carcinoma|Cancer of the Salivary Gland|Lung Cancer|Breast Cancer|Gastric Cancer|Esophageal Cancer|Colorectal Cancer|Pancreatic Adenocarcinoma|Solid Tumor	BIOLOGICAL: CAdVEC	PHASE1	INTERVENTIONAL
NCT03747744	Intratumoral Injection of Autologous CD1c (BDCA-1)+ Myeloid Dendritic Cells Plus Talimogene Laherparepvec (T-VEC)	ACTIVE_NOT_RECRUITING	Melanoma	OTHER: CD1c (BDCA-1)+ myDC	PHASE1	INTERVENTIONAL
NCT03767348	Study of RP1 Monotherapy and RP1 in Combination With Nivolumab	RECRUITING	Cancer|Melanoma (Skin)|Mismatch Repair Deficiency|Microsatellite Instability|Non-melanoma Skin Cancer|Cutaneous Melanoma|NSCLC	BIOLOGICAL: RP1|BIOLOGICAL: nivolumab	PHASE2	INTERVENTIONAL
NCT03773744	MG1-MAGEA3 With Ad-MAGEA3 and Pembrolizumab in Patients With Previously Treated Metastatic Melanoma or Cutaneous Squamous Cell Carcinoma	WITHDRAWN	Metastatic Melanoma|Squamous Cell Skin Carcinoma	DRUG: Ad-MAGEA3|DRUG: MG1-MAGEA3|DRUG: Pembrolizumab|DRUG: Cyclophosphamide	PHASE1	INTERVENTIONAL
NCT03852511	First in Human Study of NG-350A (an Oncolytic Adenoviral Vector Which Expresses an Anti-CD40 Antibody)	COMPLETED	Metastatic Cancer|Epithelial Tumor	BIOLOGICAL: NG-350A	PHASE1	INTERVENTIONAL
NCT03866525	OH2 Oncolytic Viral Therapy in Solid Tumors	RECRUITING	Solid Tumor|Gastrointestinal Cancer	BIOLOGICAL: OH2 injection, with or without irinotecan or HX008	PHASE1|PHASE2	INTERVENTIONAL
NCT03889275	A Study of MEDI5395 in Combination With Durvalumab in Participants With Select Advanced Solid Tumors	COMPLETED	Advanced Solid Tumors	BIOLOGICAL: MEDI5395|BIOLOGICAL: Durvalumab	PHASE1	INTERVENTIONAL
NCT03896568	MSC-DNX-2401 in Treating Patients With Recurrent High-Grade Glioma	RECRUITING	IDH1 wt Allele|Recurrent Anaplastic Astrocytoma|Recurrent Glioblastoma|Recurrent Gliosarcoma|Recurrent Malignant Glioma	BIOLOGICAL: Oncolytic Adenovirus Ad5-DNX-2401|PROCEDURE: Therapeutic Conventional Surgery	PHASE1	INTERVENTIONAL

NCT03911388	HSV G207 in Children With Recurrent or Refractory Cerebellar Brain Tumors	RECRUITING	Neoplasms, Brain|Glioblastoma Multiforme|Glioblastoma of Cerebellum|Neoplasms|Astrocytoma|Astrocytoma, Cerebellar|Neuroectodermal Tumors|Neuroectodermal Tumors, Primitive|Cerebellar PNET, Childhood|Cerebellar Neoplasms|Cerebellar Neoplasms, Primary|Cerebellar Neoplasm, Malignant|Cerebellar Neoplasm Malignant Primary|Neoplasm Metastases|Neoplasm Malignant|Neoplasms, Neuroepithelial|Neoplasms, Germ Cell and Embryonal|Neoplasms by Histologic Type|Neoplasms, Glandular and Epithelial|Neoplasms, Nerve Tissue|Central Nervous System Neoplasms, Primary|Central Nervous System Neoplasms, Malignant|Nervous System Neoplasms|Neoplasms by Site|Brain Diseases|Central Nervous System Diseases|Nervous System Diseases|Medulloblastoma Recurrent|HSV|Virus|Pediatric Brain Tumor|Nervous System Cancer|Primitive Neuroectodermal Tumor (PNET) of Cerebellum	BIOLOGICAL: G207	PHASE1	INTERVENTIONAL
NCT03916510	Chemoradiation With Enadenotucirev as a Radiosensitiser in Locally Advanced Rectal Cancer	COMPLETED	Locally Advanced Rectal Cancer	BIOLOGICAL: Enadenotucirev|DRUG: Capecitabine|RADIATION: Radiotherapy	PHASE1	INTERVENTIONAL
NCT03954067	A Study of an Intratumoral Oncolytic Virus in Patients With Advanced Metastatic Solid Tumors	ACTIVE_NOT_RECRUITING	Metastatic Cancer|Solid Tumors|Advanced Cancer	BIOLOGICAL: ASP9801|COMBINATION_PRODUCT: Pembrolizumab	PHASE1	INTERVENTIONAL
NCT04050436	Study Evaluating Cemiplimab Alone and Combined With RP1 in Treating Advanced Squamous Skin Cancer	ACTIVE_NOT_RECRUITING	Cutaneous Squamous Cell Carcinoma|Advanced Cutaneous Squamous Cell Carcinoma|Metastatic Cutaneous Squamous Cell Carcinoma	DRUG: Cemiplimab|BIOLOGICAL: RP1	PHASE2	INTERVENTIONAL
NCT04065152	Phase II Multicenter Study of Talimogene Laherparepvec in Classic or Endemic Kaposi Sarcoma	RECRUITING	Kaposi Sarcoma	DRUG: Talimogene laherparepvec	PHASE2	INTERVENTIONAL
NCT04097002	First in Man Clinical Study to Evaluate Safety and Tolerability of an Oncolytic Adenovirus in Prostate Cancer Patients.	RECRUITING	Adenocarcinoma of the Prostate	BIOLOGICAL: ORCA-010	PHASE1|PHASE2	INTERVENTIONAL
NCT04102618	A Window-of-opportunity Study of Pelareorep in Early Breast Cancer	TERMINATED	Breast Cancer	BIOLOGICAL: Pelareorep|DRUG: Letrozole|DRUG: Atezolizumab|DRUG: Trastuzumab	EARLY_PHASE1	INTERVENTIONAL
NCT04123470	A Phase I/II Trial Investigating LOAd703 in Combination With Atezolizumab in Malignant Melanoma	ACTIVE_NOT_RECRUITING	Malignant Melanoma	GENETIC: delolimogene mupadenorepvec|BIOLOGICAL: atezolizumab	PHASE1|PHASE2	INTERVENTIONAL
NCT04185311	Ipilimumab, Nivolumab, and Talimogene Laherparepvec Before Surgery in Treating Participants With Localized, Triple-Negative or Estrogen Receptor Positive, HER2 Negative Breast Cancer-deleted	TERMINATED	Anatomic Stage 0 Breast Cancer AJCC v8|Anatomic Stage I Breast Cancer AJCC v8|Anatomic Stage IA Breast Cancer AJCC v8|Anatomic Stage IB Breast Cancer AJCC v8|Anatomic Stage II Breast Cancer AJCC v8|Anatomic Stage IIA Breast Cancer AJCC v8|Anatomic Stage IIB Breast Cancer AJCC v8|Anatomic Stage IIIA Breast Cancer AJCC v8|Anatomic Stage IIIB Breast Cancer AJCC v8|HER2/Neu Negative|Invasive Ductal Carcinoma, Not Otherwise Specified|Progesterone Receptor Negative|Prognostic Stage 0 Breast Cancer AJCC v8|Prognostic Stage I Breast Cancer AJCC v8|Prognostic Stage IA Breast Cancer AJCC v8|Prognostic Stage IB Breast Cancer AJCC v8|Prognostic Stage II Breast Cancer AJCC v8|Prognostic Stage IIA Breast Cancer AJCC v8|Prognostic Stage IIB Breast Cancer AJCC v8|Prognostic Stage IIIA Breast Cancer AJCC v8|Prognostic Stage IIIB Breast Cancer AJCC v8|Triple-Negative Breast Carcinoma	BIOLOGICAL: Ipilimumab|BIOLOGICAL: Nivolumab|BIOLOGICAL: Talimogene Laherparepvec	PHASE1	INTERVENTIONAL
NCT04195373	A Safety Study of TMV-018 in Patients With Tumors of the Gastrointestinal Tract	WITHDRAWN	Gastrointestinal Cancer	BIOLOGICAL: TMV-018 + 5-FC|BIOLOGICAL: TMV-018 + anti-PD-1|BIOLOGICAL: TMV-018 + 5-FC + anti-PD-1	PHASE1	INTERVENTIONAL
NCT04215146	A Study to Assess Overall Response Rate by Inducing an Inflammatory Phenotype in Metastatic BReast cAnCEr With the Oncolytic Reovirus PeLareorEp in CombinaTion With Anti-PD-L1 Avelumab and Paclitaxel - BRACELET-1 Study	ACTIVE_NOT_RECRUITING	Breast Cancer Metastatic	DRUG: Paclitaxel|BIOLOGICAL: Pelareorep|DRUG: Avelumab	PHASE2	INTERVENTIONAL
NCT04217473	TNFalpha and Interleukin 2 Coding Oncolytic Adenovirus TILT-123 During TIL Treatment of Advanced Melanoma	RECRUITING	Metastatic Melanoma	BIOLOGICAL: TILT-123	PHASE1	INTERVENTIONAL
NCT04226066	Safety, Tolerability and Pharmacokinetics Characteristics of Recombinant Oncolytic Vaccinia Virus Injection T601 as a Single Drug or in Combination With Oral Flucytosine (5-FC), in Patients With Advanced Malignant Solid Tumors	UNKNOWN	Advanced Malignant Solid Tumors	BIOLOGICAL: T601|COMBINATION_PRODUCT: T601+5-FC	PHASE1|PHASE2	INTERVENTIONAL
NCT04301011	Study of TBio-6517 Given Alone or in Combination With Pembrolizumab in Solid Tumors	ACTIVE_NOT_RECRUITING	Solid Tumor|Microsatellite Stable Colorectal Cancer|HPV Positive Oropharyngeal Squamous Cell Carcinoma|Cervical Cancer|Melanoma (Skin)|Cutaneous Squamous Cell Carcinoma|Mesothelioma|Renal Cell Carcinoma|Oropharynx Cancer	BIOLOGICAL: TBio-6517|BIOLOGICAL: Pembrolizumab	PHASE1|PHASE2	INTERVENTIONAL
NCT04348916	Study of ONCR-177 Alone and in Combination With PD-1 Blockade in Adult Subjects With Advanced and/or Refractory Cutaneous, Subcutaneous or Metastatic Nodal Solid Tumors or With Liver Metastases of Solid Tumors	TERMINATED	Cancer|Melanoma|Solid Tumor|Squamous Cell Carcinoma of Head and Neck|Breast Cancer|Advanced Solid Tumor|Triple Negative Breast Cancer|Colorectal Carcinoma|Non-melanoma Skin Cancer|Liver Metastases	BIOLOGICAL: ONCR-177|BIOLOGICAL: pembrolizumab	PHASE1	INTERVENTIONAL
NCT04349436	A Phase 1B/2 Study of RP1 in Solid Organ Transplant Patients With Advanced Cutaneous Malignancies	RECRUITING	Cutaneous Squamous Cell Carcinoma|Merkel Cell Carcinoma|Basal Cell Carcinoma|Melanoma	BIOLOGICAL: RP1, intra-tumoral injection, oncolytic virus	PHASE1|PHASE2	INTERVENTIONAL
NCT04386967	OH2 Injection in Solid Tumors	RECRUITING	Solid Tumor|Melanoma	BIOLOGICAL: OH2 injection|DRUG: Keytruda	PHASE1|PHASE2	INTERVENTIONAL
NCT04445844	INCMGA00012 and Pelareorep for the Treatment of Metastatic Triple Negative Breast Cancer, IRENE Study	RECRUITING	Anatomic Stage IV Breast Cancer AJCC v8|Locally Advanced Breast Carcinoma|Metastatic Triple-Negative Breast Carcinoma|Prognostic Stage IV Breast Cancer AJCC v8|Triple-negative Breast Cancer	BIOLOGICAL: Pelareorep|OTHER: Quality-of-Life Assessment|OTHER: Questionnaire Administration|BIOLOGICAL: Retifanlimab	PHASE2	INTERVENTIONAL
NCT04482933	HSV G207 With a Single Radiation Dose in Children With Recurrent High-Grade Glioma	NOT_YET_RECRUITING	Neoplasms|High Grade Glioma|Glioblastoma Multiforme|Malignant Glioma of Brain|Anaplastic Astrocytoma of Brain|High-grade Glioma|Anaplastic Glioma|Giant Cell Glioblastoma	DRUG: Biological G207	PHASE2	INTERVENTIONAL
NCT04521764	A Vaccine (MV-s-NAP) for the Treatment of Patients With Invasive Metastatic Breast Cancer	RECRUITING	Anatomic Stage IV Breast Cancer AJCC v8|Invasive Breast Carcinoma|Metastatic Breast Adenocarcinoma|Recurrent Breast Carcinoma	BIOLOGICAL: Oncolytic Measles Virus Encoding Helicobacter pylori Neutrophil-activating Protein	PHASE1	INTERVENTIONAL
NCT04616443	OH2 Injection in Combination With HX008 for Melanoma.	RECRUITING	Melanoma	BIOLOGICAL: OH2 injection|BIOLOGICAL: HX008 injection	PHASE1|PHASE2	INTERVENTIONAL
NCT04637698	OH2 Oncolytic Viral Therapy in Pancreatic Cancer	RECRUITING	Pancreatic Cancer	BIOLOGICAL: OH2 injection	PHASE1|PHASE2	INTERVENTIONAL
NCT04665362	IIT Study of M1-c6v1 Combined With SHR-1210 and Apatinib in Patients With HCC	UNKNOWN	Advanced/Metastatic Hepatocellular Carcinoma	DRUG: Recombinant oncolytic virus M1, anti PD-1 antibody, Apatinib	PHASE1	INTERVENTIONAL
NCT04673942	A First in Human Study of AdAPT-001 in Subjects With Refractory Solid Tumors	RECRUITING	Solid Tumor, Adult|Cancer|Neoplasms	BIOLOGICAL: AdAPT-001|DRUG: Checkpoint Inhibitor, Immune	PHASE1|PHASE2	INTERVENTIONAL
NCT04685499	Phase 2 Study of OBP-301 (Telomelysin™) in Combination With Pembrolizumab and SBRT in Patients With HNSCC With Inoperable, Recurrent or Progressive Disease	TERMINATED	Head and Neck Squamous Cell Carcinoma With Inoperable Recurrent or Progressive Disease	DRUG: OBP-301|DRUG: Pembrolizumab	PHASE2	INTERVENTIONAL
NCT04695327	TNFα and IL-2 Coding Oncolytic Adenovirus TILT-123 Monotherapy	RECRUITING	Solid Tumor	BIOLOGICAL: TILT-123	PHASE1	INTERVENTIONAL
NCT04725331	A Clinical Trial Assessing BT-001 Alone and in Combination With Pembrolizumab in Metastatic or Advanced Solid Tumors	RECRUITING	Solid Tumor, Adult|Metastatic Cancer|Soft Tissue Sarcoma|Merkel Cell Carcinoma|Melanoma|Triple Negative Breast Cancer|Non Small Cell Lung Cancer	BIOLOGICAL: BT-001|BIOLOGICAL: Pembrolizumab [Keytruda]	PHASE1|PHASE2	INTERVENTIONAL
NCT04735978	Study of RP3 Monotherapy and RP3 in Combination With Nivolumab in Patients With Solid Tumours	RECRUITING	Advanced Solid Tumor	BIOLOGICAL: RP3|BIOLOGICAL: Nivolumab	PHASE1	INTERVENTIONAL
NCT04755543	A Study of LP002 for the Treatment of Patients With Malignant Digestive System Neoplasms	UNKNOWN	Digestive System Neoplasms	DRUG: LP002|DRUG: Cisplatin|DRUG: Fluorouracil|BIOLOGICAL: OH2 oncolytic virus	PHASE1	INTERVENTIONAL
NCT04758533	Clinical Trial to Assess the Safety and Efficacy of AloCELYVIR With Newly Diagnosed Diffuse Intrinsic Pontine Glioma (DIPG) in Combination With Radiotherapy or Medulloblastoma in Monotherapy	RECRUITING	Diffuse Intrinsic Pontine Glioma|Medulloblastoma, Childhood, Recurrent	BIOLOGICAL: AloCELYVIR	PHASE1|PHASE2	INTERVENTIONAL
NCT04771676	Intraperitoneal Injection of Oncolytic Viruses H101 for Patients With Refractory Malignant Ascites	COMPLETED	Refractory Malignant Ascites	DRUG: Oncorine (H101)	PHASE2	INTERVENTIONAL

NCT04787003	Oncolytic Virus (OVV-01) Injection in the Treatment of Patients With Advanced Solid Tumors	RECRUITING	Neoplasms	DRUG: oncolytic virus (OVV-01) injection	PHASE1	INTERVENTIONAL
NCT04887025	Exploratory Study of a Novel Oncolytic Vaccinia Virus RGV004 in the Treatment of Refractory/Relapsed B-cell Lymphoma	RECRUITING	Relapsed or Refractory B-cell Lymphoma	BIOLOGICAL: RGV004	PHASE1	INTERVENTIONAL
NCT05047276	Phase I/II Study of AloCelyvir in Patients With Metastatic Uveal Melanoma	NOT_YET_RECRUITING	Uveal Melanoma, Metastatic	BIOLOGICAL: AloCelyvir	PHASE1|PHASE2	INTERVENTIONAL
NCT05051696	Intra-tumor Injection of Oncolytic Viruses H101 Combined With or Without Radiotherapy in Refractory/Recurrent Gynecological Malignancies	NROLLING_BY_INVITATIO	Genital Neoplasms, Female	DRUG: H101	NA	INTERVENTIONAL
NCT05061537	Study of PF-07263689 in Participants With Selected Advanced Solid Tumors	TERMINATED	Renal Cell Cancer|Melanoma|Non-Small-Cell Lung Cancer|Hepatocellular Cancer|Bladder Cancer|Sarcoma|Head and Neck Cancer|Colorectal Cancer|Ovarian Cancer|Squamous Cell Carcinoma	BIOLOGICAL: PF-07263689|BIOLOGICAL: Sasanlimab	PHASE1	INTERVENTIONAL
NCT05068453	Study of Oncolytic Virus in Combination With HX-008 and Radiotherapy in Melanoma Patients With Liver Metastasis	NOT_YET_RECRUITING	Melanoma Stage IV	DRUG: Recombinant Oncolytic HSV-2 Therapeutic Injecta (Vero Cell) for Human Use (rHSV2hGM-CSF)|DRUG: Recombinant humanized anti-PD-1 monoclonal antibody for injection|RADIATION: RT	PHASE1	INTERVENTIONAL
NCT05070221	Study of Oncolytic Virus in Combination With HX-008 and Axitinib in Melanoma Patients With Liver Metastasis	NOT_YET_RECRUITING	Melanoma Stage IV	DRUG: Recombinant Oncolytic HSV-2 Therapeutic Injecta (Vero Cell) for Human Use (rHSV2hGM-CSF)|DRUG: Recombinant humanized anti-PD-1 monoclonal antibody for injection|DRUG: Axitinib	PHASE1	INTERVENTIONAL
NCT05076760	Study of MEM-288 Oncolytic Virus in Solid Tumors Including Non-Small Cell Lung Cancer (NSCLC)	RECRUITING	Solid Tumor|Advanced Cancer|Metastatic Cancer|Non Small Cell Lung Cancer|Cutaneous Squamous Cell Carcinoma|Merkel Cell Carcinoma|Melanoma|Pancreatic Cancer|Triple Negative Breast Cancer|Head and Neck Cancer	BIOLOGICAL: MEM-288 Intratumoral Injection	PHASE1	INTERVENTIONAL
NCT05081492	CF33-hNIS-antiPDL1 for the Treatment of Metastatic Triple Negative Breast Cancer	RECRUITING	Anatomic Stage IV Breast Cancer AJCC v8|Metastatic Triple-Negative Breast Carcinoma|Prognostic Stage IV Breast Cancer AJCC v8	BIOLOGICAL: Oncolytic Virus CF33-expressing hNIS/Anti-PD-L1 Antibody	PHASE1	INTERVENTIONAL
NCT05084430	Study of Pembrolizumab and M032 (NSC 733972)	RECRUITING	Glioblastoma Multiforme|Anaplastic Astrocytoma|Gliosarcoma	DRUG: M032|DRUG: Pembrolizumab	PHASE1|PHASE2	INTERVENTIONAL
NCT05113290	Effect and Safety of Recombinant Human Adenovirus Type 5 in Advanced HCC With Stable Disease After Sorafenib Treatment	ACTIVE_NOT_RECRUITING	Hepatocellular Carcinoma	DRUG: Recombinant Human Adenovirus Type 5 injection|DRUG: Sorafenib	PHASE4	INTERVENTIONAL
NCT05122572	RT-01 Monotherapy and in Combination With Nivolumab in Patients With Advanced Solid Tumors	RECRUITING	Advanced Solid Tumor	BIOLOGICAL: Oncolytic Virus Injection (RT-01)	PHASE1	INTERVENTIONAL
NCT05124002	Recombinant Human Adenovirus Type 5 Plus HAIC of FOLFOX for Intrahepatic Cholangiocarcinoma	NOT_YET_RECRUITING	Cholangiocarcinoma, Intrahepatic	DRUG: Recombinant Human Adenovirus Type 5|DRUG: HAIC of FOLFOX	PHASE4	INTERVENTIONAL
NCT05136937	A Clinical Study of Intratumoral Administration of RT-01 in Patients With Advanced Solid Tumors	RECRUITING	Solid Tumor|Advanced Cancer	BIOLOGICAL: Oncolytic Virus Injection(RT-01)	PHASE1	INTERVENTIONAL
NCT05139056	Multiple Doses of Neural Stem Cell Virotherapy (NSC-CRAd-S-pk7) for the Treatment of Recurrent High-Grade Gliomas	RECRUITING	Recurrent Anaplastic Astrocytoma|Recurrent Anaplastic Oligoastrocytoma|Recurrent Anaplastic Oligodendroglioma|Recurrent Glioblastoma|Recurrent Gliosarcoma|Recurrent Malignant Glioma|Recurrent WHO Grade II Glioma|Recurrent WHO Grade III Glioma	BIOLOGICAL: Neural Stem Cells-expressing CRAd-S-pk7|PROCEDURE: Resection	PHASE1	INTERVENTIONAL
NCT05180851	Safety and Efficacy of Recombinant Oncolytic Adenovirus L-IFN Injection in Relapsed/Refractory Solid Tumors Clinical Study	RECRUITING	Head and Neck Cancer|Melanoma|Breast Cancer|Bladder Cancer|Ovarian Carcinoma|Cervical Carcinoma|Lung Cancer	DRUG: Recombinant L-IFN adenovirus injection	EARLY_PHASE1	INTERVENTIONAL
NCT05205408	A Study of Intratumoral Administration of Oncolytic Virus Injection (RT-01) in Patients With Advanced Solid Tumors	RECRUITING	Advanced Solid Tumor	BIOLOGICAL: Oncolytic Virus Injection(RT-01)	PHASE1	INTERVENTIONAL
NCT05205421	A Study of Oncolytic Virus Injection (RT-01) in Patients With Extensive-Stage Small Cell Lung Cancer	RECRUITING	Advanced Solid Tumor	BIOLOGICAL: Oncolytic Virus Injection(RT-01)	PHASE1	INTERVENTIONAL
NCT05222932	Oncolytic Adenovirus TILT-123 and Avelumab for Treatment of Solid Tumors Refractory to or Progressing After Anti-PD(L)1	RECRUITING	Melanoma|Head and Neck Squamous Cell Carcinoma	BIOLOGICAL: TILT-123|DRUG: Avelumab	PHASE1	INTERVENTIONAL
NCT05228119	A Study of Oncolytic Virus Injection (RT-01) in Combination With PD-1 Inhibitor in Patients With Advanced Solid Tumors	RECRUITING	Advanced Solid Tumor	BIOLOGICAL: Oncolytic Virus Injection(RT-01)	PHASE1	INTERVENTIONAL
NCT05232136	OH2 Oncolytic Viral Therapy in Non-Muscle-Invasive Bladder Cancer	RECRUITING	Non-muscle-invasive Bladder Cancer	BIOLOGICAL: OH2 injection	PHASE1|PHASE2	INTERVENTIONAL
NCT05234905	H101 Combined With Camrelizumab for Recurrent Cervical Cancer	NOT_YET_RECRUITING	Uterine Cervical Neoplasms|Oncolytic Virotherapy|Camrelizumab	DRUG: Recombinant human adenovirus type 5＋Camrelizumab	PHASE2	INTERVENTIONAL
NCT05235074	OH2 Oncolytic Viral Therapy in Central Nervous System Tumors	RECRUITING	Central Nervous System Tumors	BIOLOGICAL: OH2 injection	PHASE1|PHASE2	INTERVENTIONAL
NCT05248789	OH2 Oncolytic Viral Therapy in Advanced Bladder Cancer	RECRUITING	Advanced Bladder Carcinoma	BIOLOGICAL: OH2 injection	PHASE2	INTERVENTIONAL
NCT05271279	A Study of OVV-01 Injection in Combination With IBR900 Cell Injection in Patients With Advanced Malignant Tumors	TERMINATED	Advanced Solid Tumor|Relapsed/Refractory Lymphoma	BIOLOGICAL: OVV-01 Injection+IBR900 Cell Injection	EARLY_PHASE1	INTERVENTIONAL
NCT05271318	Oncolytic Adenovirus TILT-123 With Pembrolizumab as Treatment for Ovarian Cancer	RECRUITING	Platinum-refractory Ovarian Carcinoma|Platinum-resistant Ovarian Cancer|Platinum-Resistant Fallopian Tube Carcinoma|Platinum-Resistant Primary Peritoneal Carcinoma|Platinum-Refractory Fallopian Tube Carcinoma|Platinum-Refractory Primary Peritoneal Carcinoma|Platinum-Sensitive Ovarian Cancer in Which the Participant Has Allergy or Severe Intolerance to Carboplatin and/or Cisplatin|Platinum-Sensitive Fallopian Tube Carcinoma in Which the Participant Has Allergy or Severe Intolerance to Carboplatin and/or Cisplatin|Platinum-Sensitive Primary Peritoneal Carcinoma in Which the Participant Has Allergy or Severe Intolerance to Carboplatin and/or Cisplatin	BIOLOGICAL: TILT-123|BIOLOGICAL: Pembrolizumab	PHASE1	INTERVENTIONAL
NCT05281471	Efficacy & Safety of Olvi-Vec and Platinum-doublet + Bevacizumab Compared to Platinum-doublet + Bevacizumab in Platinum-Resistant/Refractory Ovarian Cancer (OnPrime, GOG-3076)	RECRUITING	Platinum-resistant Ovarian Cancer|Platinum-refractory Ovarian Cancer|Fallopian Tube Cancer|Primary Peritoneal Cancer|High-grade Serous Ovarian Cancer|Endometrioid Ovarian Cancer|Ovarian Clear Cell Carcinoma	BIOLOGICAL: olvimulogene nanivacirepvec|DRUG: Platinum chemotherapy: carboplatin (preferred) or cisplatin|DRUG: Non-platinum chemotherapy: Physician's Choice of gemcitabine, taxane (paclitaxel, docetaxel or nab-paclitaxel) or pegylated liposomal doxorubicin|DRUG: Bevacizumab (or biosimilar)	PHASE3	INTERVENTIONAL
NCT05346484	A Study of CF33-hNIS (VAXINIA), an Oncolytic Virus, as Monotherapy or in Combination With Pembrolizumab in Adults With Metastatic or Advanced Solid Tumors	RECRUITING	Solid Tumor|Solid Carcinoma|Solid Tumor, Adult|Metastatic Cancer|Advanced Solid Tumor	BIOLOGICAL: CF33-hNIS|BIOLOGICAL: Pembrolizumab	PHASE1	INTERVENTIONAL
NCT05361954	Study to Assess the Safety and Preliminary Efficacy of STI-1386 Oncolytic Virus in Relapsed or Refractory Solid Tumors	NOT_YET_RECRUITING	Cancer|Cancer of Pancreas|Sarcoma|Hepatic Metastasis|Solid Tumor	DRUG: STI-1386	PHASE1	INTERVENTIONAL
NCT05376527	Open Multi-cohort Study of the First Phase of Safety of a Drug Based on Double Recombinant Vaccinia Virus VV-GMCSF-Lact	RECRUITING	Oncolytic Virotherapy	BIOLOGICAL: Double Recombinant Vaccinia Virus VV-GMCSF-Lact	PHASE1	INTERVENTIONAL
NCT05387226	Intravenous Injection of Oncolytic Virus Injection (RT-01) in Patients With Relapsed or Refractory T-cell Lymphoma	NOT_YET_RECRUITING	T-cell Lymphoma|Virus|Intravenous Injection	BIOLOGICAL: Oncolytic Virus Injection(RT-01)	PHASE1	INTERVENTIONAL
NCT05393440	First-in-human (FIH) Phase I Trial of BS-006 in Cervical Cancer	RECRUITING	Uterine Cervical Neoplasms	BIOLOGICAL: BS-006	PHASE1	INTERVENTIONAL
NCT05427487	Study of Intratumoral IVX037 in Patients With Advanced or Metastatic Solid Tumours	RECRUITING	Colorectal Cancer|Gastroesophageal Cancer|Ovarian Cancer	BIOLOGICAL: IVX037	PHASE1	INTERVENTIONAL
NCT05477849	A Clinical Study to Evaluate the Safety, Tolerability, Pharmacokinetics and Biologic Effect of VG2025 in Patients With Solid Tumors	RECRUITING	Advanced Malignant Solid Tumor	DRUG: Recombinant Human IL12/15 Dual-Regulated Oncolytic HSV-1 Injection	PHASE1	INTERVENTIONAL
NCT05561491	A Study of ONCOS-102 in Combination With Other Novel Immune-therapies in Advanced Treatment-resistant Melanoma Patients	WITHDRAWN	Melanoma	BIOLOGICAL: ONCOS-102|BIOLOGICAL: Balstilimab	PHASE2	INTERVENTIONAL
NCT05564897	Oncolytic Adenovirus Combined With PD-1 Inhibitor in Patients With Non-muscle-invasive Bladder Cancer	RECRUITING	Bladder Cancer	DRUG: H101, Camrelizumab	PHASE2	INTERVENTIONAL
NCT05598268	A Study of T3011 Administered Via Intravenously in Patients With Advanced Solid Tumors.	RECRUITING	Advanced Solid Tumor|Lung Cancer|Liver Cancer|Lymphoma|Mesothelioma of Pleura	BIOLOGICAL: T3011	PHASE1|PHASE2	INTERVENTIONAL
NCT05600582	A Study of CodaLytic, an Intratumoral Oncolytic Virus, in Patients With Breast Cancer	NOT_YET_RECRUITING	Breast Neoplasms|Neoplasm Metastasis	BIOLOGICAL: CodaLytic	PHASE1	INTERVENTIONAL
NCT05602792	A Study of T3011 Administered Via Intratumoral Injection in Patients With Advanced Solid Tumors	RECRUITING	Advanced Solid Tumor|Sarcoma|HNSCC|Breast Cancer|Esophagus Cancer|NSCLC|Non-melanoma Skin Cancer	BIOLOGICAL: T3011|BIOLOGICAL: T3011|BIOLOGICAL: T3011|BIOLOGICAL: T3011|BIOLOGICAL: T3011|BIOLOGICAL: T3011|BIOLOGICAL: T3011	PHASE1|PHASE2	INTERVENTIONAL
NCT05644509	Study on the Treatment of Advanced Malignant Solid Tumor With Revottack and PD-1 Inhibitor	NOT_YET_RECRUITING	Advanced Solid Tumor	BIOLOGICAL: Oncolytic Virus Injection（Revottack）+PD-1	PHASE1	INTERVENTIONAL
NCT05648006	First-line Maintenance of OH2 Injection for Advanced Colorectal Cancer	NOT_YET_RECRUITING	Advanced Colorectal Carcinoma	BIOLOGICAL: OH2|DRUG: Capecitabine|DRUG: Bevacizumab	PHASE2	INTERVENTIONAL

<table>
<tr><td>NCT05673811</td><td>Study of Nab-Paclitaxel and Gemcitabine and Plus/Minus VCN-01 in Patients With Metastatic Pancreatic Cancer</td><td>RECRUITING</td><td>Pancreatic Adenocarcinoma|Metastatic</td><td>DRUG: Nab-paclitaxel|DRUG: Gemcitabine|GENETIC: VCN-01</td><td>PHASE2</td><td>INTERVENTIONAL</td></tr>
<tr><td>NCT05684731</td><td>Safety and Efficacy of KM1 in Subjects With Recurrent or Refractory Ovarian Cancer</td><td>NOT_YET_RECRUITING</td><td>Ovarian Cancer</td><td>BIOLOGICAL: KM1|DRUG: Chemotherapy</td><td>PHASE1</td><td>INTERVENTIONAL</td></tr>
<tr><td>NCT05698459</td><td>OH2 Oncolytic Viral Therapy Via Transcatheter Intraarterial Infusion in Patients With Advanced Liver Cancer</td><td>RECRUITING</td><td>Advanced Liver Cancer</td><td>BIOLOGICAL: OH2 injection</td><td>PHASE1</td><td>INTERVENTIONAL</td></tr>
<tr><td>NCT05717699</td><td>Oncolytic Virus Ad-TD-nsIL12 for Progressive Pediatric Diffuse Intrinsic Pontine Glioma</td><td>RECRUITING</td><td>Oncolytic Virus|Diffuse Intrinsic Pontine Glioma|Adverse Drug Event</td><td>BIOLOGICAL: Ad-TD-nsIL12</td><td>PHASE1</td><td>INTERVENTIONAL</td></tr>
<tr><td>NCT05717712</td><td>Oncolytic Virus Ad-TD-nsIL12 for Primary Pediatric Diffuse Intrinsic Pontine Glioma</td><td>RECRUITING</td><td>Oncolytic Virus|Diffuse Intrinsic Pontine Glioma|Adverse Drug Event</td><td>BIOLOGICAL: Ad-TD-nsIL12</td><td>PHASE1</td><td>INTERVENTIONAL</td></tr>
<tr><td>NCT05733598</td><td>RP3 in Combination With 1L or 2L Therapy in Patients With Locally Advanced Unresectable or Metastatic HCC</td><td>NOT_YET_RECRUITING</td><td>Locally Advanced Hepatocellular Carcinoma|Recurrent Hepatocellular Carcinoma|Metastatic Hepatocellular Carcinoma</td><td>BIOLOGICAL: RP3|BIOLOGICAL: atezolizumab|BIOLOGICAL: bevacizumab</td><td>PHASE2</td><td>INTERVENTIONAL</td></tr>
<tr><td>NCT05733611</td><td>RP2/RP3 in Combination With Atezolizumab and Bevacizumab for the Treatment of Patients With CRC</td><td>RECRUITING</td><td>Refractory Metastatic Colorectal Cancer|pMMR|MSS</td><td>BIOLOGICAL: RP2|BIOLOGICAL: RP3|BIOLOGICAL: atezolizumab|BIOLOGICAL: bevacizumab</td><td>PHASE2</td><td>INTERVENTIONAL</td></tr>
<tr><td>NCT05743270</td><td>Study of RP3 in Combination With Nivolumab and Other Therapy in Patients With Locoregionally Advanced or Recurrent SCCHN</td><td>NOT_YET_RECRUITING</td><td>Squamous Cell Carcinoma of Head and Neck|Locally Advanced Head and Neck Squamous Cell Carcinoma|Recurrent Head and Neck Squamous Cell Carcinoma</td><td>BIOLOGICAL: RP3|OTHER: CCRT(concurrent chemoradiation therapy)|OTHER: carboplatin and paclitaxel|BIOLOGICAL: nivolumab</td><td>PHASE2</td><td>INTERVENTIONAL</td></tr>
<tr><td>NCT05788926</td><td>A Clinical Trial of TG6050 in Patients With Metastatic Non-Small Cell Lung Cancer (Delivir)</td><td>RECRUITING</td><td>Non-small Cell Lung Cancer</td><td>DRUG: TG6050</td><td>PHASE1</td><td>INTERVENTIONAL</td></tr>
<tr><td>NCT05801783</td><td>A Clinical Study on Oncolytic Virus Injection (R130) for the Treatment of Relapsed/Refractory Ovarian Cancer</td><td>RECRUITING</td><td>Ovarian Cancer|Peritoneal Carcinomatosis|Fallopian Tube Cancer</td><td>DRUG: Recombinant oncolytic herpes simplex virus type 1 (R130)</td><td>EARLY_PHASE1</td><td>INTERVENTIONAL</td></tr>
<tr><td>NCT05812677</td><td>A Clinical Study on Oncolytic Virus Injection (R130) for the Treatment of Relapsed/Refractory Cervical and Endometrial Cancer</td><td>RECRUITING</td><td>Cervical Cancer|Endometrial Cancer|Advanced Cancer</td><td>DRUG: Recombinant oncolytic herpes simplex virus type I (R130)</td><td>EARLY_PHASE1</td><td>INTERVENTIONAL</td></tr>
<tr><td>NCT05830240</td><td>A Clinical Study on Oncolytic Virus Injection (R130 OV) for the Treatment of Relapsed/Refractory Head and Neck Cancer</td><td>RECRUITING</td><td>Head and Neck Cancer|Esophageal Cancer|Otorhinolaryngologic Neoplasms|Ear Cancer|Nose Cancer|Laryngeal Cancer|Pharyngeal Cancer</td><td>DRUG: Recombinant oncolytic herpes simplex virus type 1 (R130)</td><td>EARLY_PHASE1</td><td>INTERVENTIONAL</td></tr>
<tr><td>NCT05851456</td><td>A Clinical Study on Oncolytic Virus Injection (R130) for the Treatment of Relapsed/Refractory Bone and Soft Tissue Tumors</td><td>RECRUITING</td><td>Osteosarcoma|Sarcoma|Sarcoma,Soft Tissue|Bone Tumor</td><td>DRUG: Recombinant oncolytic herpes simplex virus type I (R130)</td><td>EARLY_PHASE1</td><td>INTERVENTIONAL</td></tr>
<tr><td>NCT05860374</td><td>A Clinical Study on Oncolytic Virus Injection (R130) for the Treatment of Advanced Solid Tumors</td><td>RECRUITING</td><td>Sarcoma|Carcinoma|Breast Cancer|Pancreatic Cancer|Colorectal Cancer|Gastric Cancer|Liver Cancer|Lung Cancer|Gynecologic Cancer</td><td>DRUG: Recombinant oncolytic herpes simplex virus type 1 (R130)</td><td>EARLY_PHASE1</td><td>INTERVENTIONAL</td></tr>
<tr><td>NCT05868707</td><td>OH2 Injection in Melanoma</td><td>RECRUITING</td><td>Melanoma</td><td>DRUG: OH2|DRUG: Salvage chemotherapy or best supportive care</td><td>PHASE3</td><td>INTERVENTIONAL</td></tr>
<tr><td>NCT05886075</td><td>A Clinical Study on Oncolytic Virus Injection (R130) for the Treatment of Relapsed/Refractory Advanced Solid Tumors</td><td>RECRUITING</td><td>Lung Cancer|Bronchial Cancer|Non Small Cell Lung Cancer|Small Cell Lung Cancer|Sarcoma|Colorectal Cancer|Gastric Cancer|Liver Cancer|Breast Cancer|Pancreatic Cancer|Head and Neck Cancer|Ovarian Cancer</td><td>DRUG: Recombinant oncolytic herpes simplex virus type 1 (R130)</td><td>EARLY_PHASE1</td><td>INTERVENTIONAL</td></tr>
<tr><td>NCT05904236</td><td>Safety and Tolerability of Intravenous Administration of ICVB-1042</td><td>RECRUITING</td><td>Patients With Advanced Solid Tumors</td><td>DRUG: Treatment with ICVB-1042 administered intravenously</td><td>PHASE1</td><td>INTERVENTIONAL</td></tr>
<tr><td>NCT05914376</td><td>Safety of Recombinant Human IL-21 Oncolytic Vaccinia Virus Injection（hV01) in Advanced Tumors</td><td>RECRUITING</td><td>Advanced Solid Tumors</td><td>BIOLOGICAL: Recombinant human IL-21 oncolytic vaccinia virus injection</td><td>PHASE1</td><td>INTERVENTIONAL</td></tr>
<tr><td>NCT05927298</td><td>Province of Ontario Strategy for Personalized Management of Pancreatic Cancer Trial</td><td>RECRUITING</td><td>Pancreas Cancer</td><td>OTHER: Non-interventional</td><td></td><td>OBSERVATIONAL</td></tr>
<tr><td>NCT05928962</td><td>An Exploratory Single-arm Study: PD-1 With Recombinant Human Adenovirus Type 5 Injection for Malignant Melanomas</td><td>ENROLLING_BY_INVITATION</td><td>Malignant Melanomas</td><td>DRUG: Recombinant Human Adenovirus Type 5 Injection</td><td>PHASE1</td><td>INTERVENTIONAL</td></tr>
<tr><td>NCT05938296</td><td>oHSV2-PD-L1/CD3-BsAb Administered Via Intramural Injection</td><td>NOT_YET_RECRUITING</td><td>Solid Tumor</td><td>DRUG: BS006 Injection</td><td>PHASE1</td><td>INTERVENTIONAL</td></tr>
<tr><td>NCT05954091</td><td>OH2 Administered by Intratumoral Injection</td><td>NOT_YET_RECRUITING</td><td>Solid Tumor</td><td>DRUG: OH2 Injection</td><td>PHASE1</td><td>INTERVENTIONAL</td></tr>
<tr><td>NCT05961111</td><td>A Clinical Study on Oncolytic Virus Injection (R130 OV) for the Treatment of Advanced Solid Tumors</td><td>RECRUITING</td><td>Sarcoma|Carcinoma|Digestive Cancer|Breast Cancer|Lung Cancer|Brain Cancer|Melanoma|Gynecologic Cancer|Head and Neck Cancer|Kidney Cancer</td><td>DRUG: Recombinant oncolytic herpes simplex virus type 1 (R130)</td><td>EARLY_PHASE1</td><td>INTERVENTIONAL</td></tr>
<tr><td>NCT06008925</td><td>Clinical Study of VG161 Combined With Nivolumab Injection in Patients With Advanced Metastatic Gastric Cancer</td><td>RECRUITING</td><td>Metastatic Gastric Cancer</td><td>DRUG: Recombinant Human IL12/15-PDL1B Oncolytic HSV-1 Injection (Vero Cell))|DRUG: Nivolumab Injection</td><td>PHASE1|PHASE2</td><td>INTERVENTIONAL</td></tr>
</table>
